# Weak correlation between sequence conservation in promoter regions and in protein-coding regions of human-mouse orthologous gene pairs

**DOI:** 10.1186/1471-2164-9-152

**Published:** 2008-04-02

**Authors:** Hirokazu Chiba, Riu Yamashita, Kengo Kinoshita, Kenta Nakai

**Affiliations:** 1 Human Genome Center, Institute of Medical Science, University of Tokyo, 4-6-1 Shirokanedai, Minato-ku, Tokyo 108-8639, Japan; 2 Institute for Bioinformatics Research and Development (BIRD), Japan Science and Technology Agency (JST), Science Plaza, 5-3 Yonban-cho, Chiyoda-ku, Tokyo 102-8666, Japan

## Abstract

**Background:**

Interspecies sequence comparison is a powerful tool to extract functional or evolutionary information from the genomes of organisms. A number of studies have compared protein sequences or promoter sequences between mammals, which provided many insights into genomics. However, the correlation between protein conservation and promoter conservation remains controversial.

**Results:**

We examined promoter conservation as well as protein conservation for 6,901 human and mouse orthologous genes, and observed a very weak correlation between them. We further investigated their relationship by decomposing it based on functional categories, and identified categories with significant tendencies. Remarkably, the 'ribosome' category showed significantly low promoter conservation, despite its high protein conservation, and the 'extracellular matrix' category showed significantly high promoter conservation, in spite of its low protein conservation.

**Conclusion:**

Our results show the relation of gene function to protein conservation and promoter conservation, and revealed that there seem to be nonparallel components between protein and promoter sequence evolution.

## Background

Comparative analysis is a powerful approach to extract functional or evolutionary information from biological sequences (reviewed in [1-3]). There were many pioneering works on the molecular evolution of mammalian protein sequences [4], which were followed by large scale comparative analyses between species. Wolfe and Sharp [5] analyzed a collection of 363 mouse and rat orthologous gene pairs, and Murphy [6] examined 615 pairs of orthologous genes between human and rodents. Makalowski et al. [7] performed a comparative analysis for 1,196 cDNA pairs between human and rodents. These studies revealed that the evolutionary rates of protein sequences depend on the protein functions. For example, ribosomal proteins and Ras-like GTPases are highly conserved [7], while proteins for antimicrobial host defenses are highly divergent [6].

On the other hand, comparisons of upstream non-coding sequences have been conducted to investigate the regulatory sequences. The complete sequences of mammalian genomes [8-10] facilitated large scale comparisons of non-coding sequences, which provided insights about regulatory sequences. Iwama and Gojobori [11] compared the upstream sequences of 3,750 human-mouse orthologous gene pairs and found that transcription factor genes, particularly those related to developmental processes, show high upstream sequence conservation. Lee et al. [12] also reported that genes involved in adaptive processes tend to have highly conserved upstream regions in mammalian genomes. Choi et al. [13] investigated the levels of non-coding conservation, focusing on tissue-specific genes.

While many efforts have been made to examine protein sequence conservation or regulatory sequence conservation, the relationships between them are poorly understood. Although several researchers have addressed a similar issue, where the relationship between protein evolution and regulatory evolution was examined based on microarray expression data [14-19], there is a discrepancy among their conclusions. Some of the researchers concluded that these two kinds of evolution are decoupled [14,17], while others claimed that there was indeed a correlation between them [15,16,18,19]. Since a substantial amount of the regulatory information is embedded in the promoter region, which is located proximal to the transcriptional start site, examining the protein sequence evolution in relation to the promoter sequence is an alternative approach to address this problem. Recently, Castillo-Davis et al. [20] made the first investigation of the relationship between protein and cis-regulatory sequence evolution using nematode genomes, and observed a weak correlation. As a step to broaden our understanding of genome evolution and function, it seems important to examine these sequences in mammalian genomes, and to analyze them in detail to dissect the relationship. However, such a sequence level analysis has not been carried out for mammals. One of the main problems is the precise determination of the TSS, which is indispensable for identifying reliable promoter regions.

Experimentally validated TSS information can provide a basis for a reliable promoter analysis. Based on large-scale collections of full-length cDNAs [21-24], our group constructed DBTSS, database of transcriptional start sites [25,26], which enabled the reliable identification, annotation and analysis of promoter regions [27-29]. Since abundant TSS data for human and mouse were integrated into DBTSS, large scale cross-species comparisons of promoter regions became possible [30,31]. Recently, our group reported an updated version of DBTSS [32], in which the amount of data was significantly increased.

In this study, we compared promoter sequences as well as protein sequences for 6,901 human and mouse orthologous genes, aiming at two points. First, we carried out a comprehensive comparison of human and mouse promoter sequences, to examine the relationship between promoter conservation and gene function. Second, we tried to elucidate what kinds of relationships exist between promoter conservation and protein conservation in mammals. In the second part, we not only examined the extent of correlation between them, but also investigated the relationship in further detail, by decomposing it based on the functional categories of genes. The results revealed that there seem to be nonparallel components between protein and promoter sequence evolution.

## Results

### Promoter sequence comparison between human and mouse

**Figure 1:**
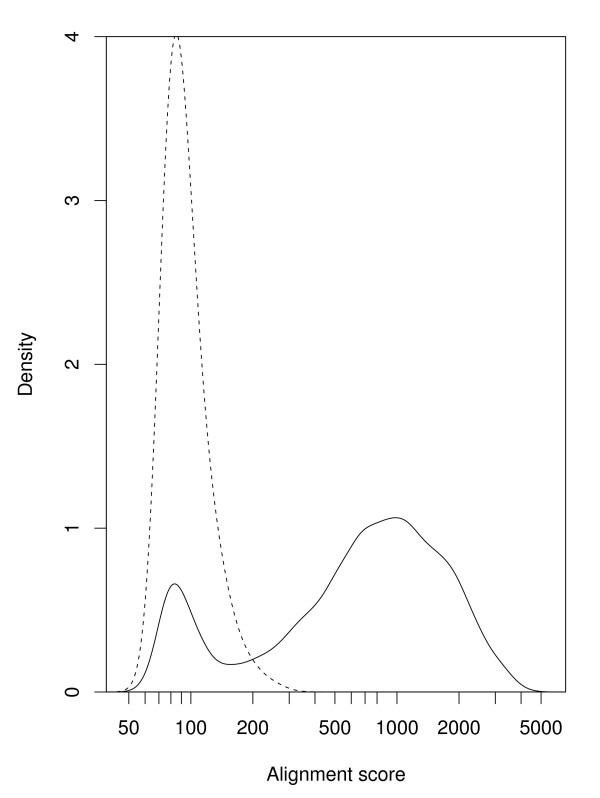
**Distribution of alignment scores of human and mouse promoters**. The distribution for the orthologous gene pairs is depicted by the solid line, and the distribution for the negative control pairs is shown by the dashed line. The x-axis is shown in a logarithmic scale.

We began the analysis with 8,429 promoter pairs of one-to-one orthologous genes between human and mouse. These pairs were compared by using the local alignment program **water **from the EMBOSS package [33]. The resulting distributions of the alignment scores are shown in Figure 1. The distribution has two peaks: a major peak around 1000, and a minor peak a little lower than 100. The minor peak corresponds to the negative control distribution created from randomly shuffled promoter pairs (depicted with a dashed line), indicating the presence of non-orthologous promoters that are not evolutionally related to each other (for an explanation of this phenomenon, see Discussion). The apparent separation of the major and minor peaks indicates that we can discriminate orthologous promoters from non-orthologous ones by examining the local alignment scores. For the following analyses, we used the 6,901 promoter pairs with alignment scores ≥ 200 (82% of the initial data set) to eliminate non-orthologous pairs. The threshold of 200 was chosen so that the proportion of non-orthologous pairs with scores over the threshold was low enough: 200 is the 1.5 percentile of the negative control distribution, and the height of the minor peak is 0.16 times that of the negative control, and thus the proportion of non-orthologous pairs with scores ≥ 200 is estimated to be 0.24% (see Additional file 1). It was possible that the offset of representative TSSs between human and mouse could bias the alignment scores. We evaluated this effect by estimating the offset from the differences in the local alignment end positions and shifting the mouse promoter as much as the offset. As a result of the promoter alignment with the offset correction, we confirmed that the bias was very small (data not shown). Therefore, we retained the original approach.

### Relationship between gene function and promoter conservation

**Figure 2:**
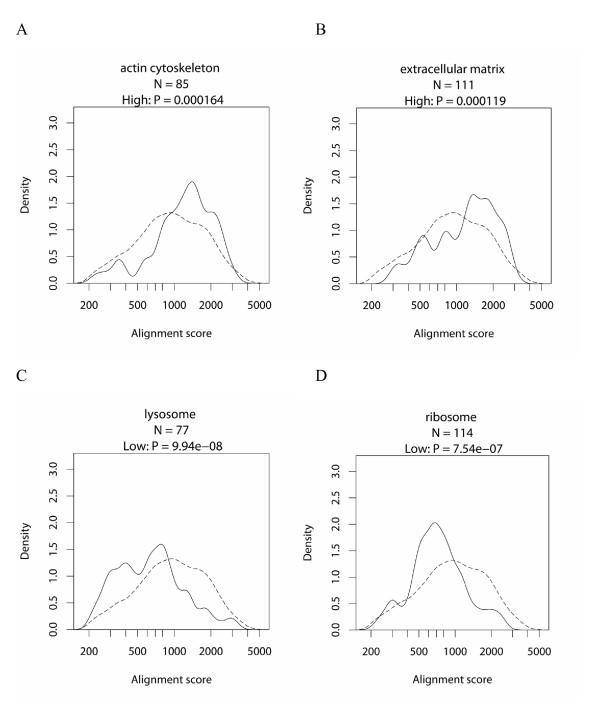
**Distribution of alignment scores of human and mouse promoters for several categories with significant tendencies**. For the high conservation tendency, actin cytoskeleton (A) and extracellular matrix (B), for the low conservation tendency, lysosome (C) and ribosome (D). For each of A-D, the solid line shows the distribution of the alignment scores for genes with the specific GO term, and the dashed line shows the distribution for the control gene set (see Materials and Methods for details).

Based on the promoter sequence comparison between human and mouse for the 6,901 genes, we investigated the relationship between gene function and promoter conservation. Annotations of genes were made by associating human genes with GO terms. To this end, we developed a slimmed-down version of the GO vocabulary, containing 52 terms for biological process (P), 22 for cellular component (C) and 26 for molecular function (F) (see Materials and Methods and Additional file 2 for details). We tested whether the alignment scores for a set of genes associated with a GO term are significantly high or low by a Wilcoxon rank sum test. The resulting GO terms with high promoter conservation are listed in Table 1, and those with low conservation are in Table 2 (only terms with a P-value < 0.01 are in the tables; for the complete list of results, see Additional file 3). Figure 2 shows the distributions of the alignment scores for several GO terms with significant tendencies (all of the distributions for the GO terms listed in Table 1 and 2 are shown in Additional file 4). When we tried the global alignment score, we obtained quite similar tendencies (data not shown). We also confirmed that eliminating the coding sequences from the promoter dataset does not significantly influence the observed tendencies (data not shown, see Materials and Methods for details).

**Table 1:** GO categories with high promoter conservation. Terms of biological process are labeled as P, cellular component as C, molecular function as F.

**GO term**	**Number of genes**	**P-value**
P:development	649	0
P:regulation of transcription	602	1.67E-15
F:transcription factor activity	263	3.44E-15
P:transcription	640	4.11E-14
P:nervous system development	154	1.99E-10
P:organ development	213	2.30E-10
P:signal transduction	994	5.19E-10
F:DNA binding	628	3.19E-08
P:morphogenesis	212	9.78E-08
P:cell surface receptor linked signal transduction	363	2.23E-06
P:negative regulation of metabolism	107	1.02E-05
F:receptor binding	221	1.90E-05
P:cell-cell signaling	176	2.27E-05
F:cytoskeletal protein binding	137	4.97E-05
P:negative regulation of biological process	327	6.87E-05
F:ion channel activity	98	9.87E-05
C:extracellular matrix	111	0.000119
C:actin cytoskeleton	85	0.000164
P:cell differentiation	173	0.000179
P:cell adhesion	242	0.000182
P:cellular morphogenesis	111	0.000607
F:ion transporter activity	237	0.001493
P:protein amino acid phosphorylation	213	0.001593
P:ion transport	239	0.001825
F:protein kinase activity	220	0.002033
P:intracellular signaling cascade	431	0.006872
P:chromosome organization and biogenesis	105	0.007832
C:plasma membrane	608	0.008026

**Table 2:** GO categories with low promoter conservation. Terms of biological process are labeled as P, cellular component as C, molecular function as F.

**GO term**	**Number of genes**	**P-value**
C:mitochondrion	398	5.31E-09
F:oxidoreductase activity	309	2.07E-08
C:lysosome	77	9.94E-08
C:ribosome	114	7.54E-07
P:lipid metabolism	260	1.04E-06
P:carboxylic acid metabolism	225	4.43E-06
F:structural constituent of ribosome	130	5.76E-06
P:amino acid metabolism	112	0.000102
P:electron transport	151	0.000236
P:catabolism	260	0.000251
P:carbohydrate metabolism	220	0.000278
C:peroxisome	49	0.000623
P:protein biosynthesis	283	0.00063
F:nuclease activity	60	0.000772
P:response to biotic stimulus	318	0.000893
C:nucleolus	63	0.004455
P:immune response	270	0.005437
F:iron ion binding	111	0.0055
F:peptidase activity	227	0.005592
P:proteolysis	259	0.006844

In Table 1, we confirmed that the most significant terms are P:development and P:regulation of transcription [11,12]. Furthermore, an overall observation of the table revealed that the terms with high promoter conservation are related to signaling events inside as well as outside of the cell (P:cell-cell signaling, P:cell surface receptor linked signal transduction, P:ion transport, and P:intracellular signaling cascade). On the other hand, Table 2 covers a wide range of metabolism (P:lipid metabolism, P:carbohydrate metabolism, P:protein biosynthesis, P:proteolysis, P:electron transport, F:oxidoreductase activity, F:nuclease activity). Table 2 also contains cellular components, such as C:mitochondrion, C:lysosome, C:ribosome and C:peroxisome, which correspond to the metabolism-related terms.

### Relationship between gene function and protein conservation

**Figure 3:**
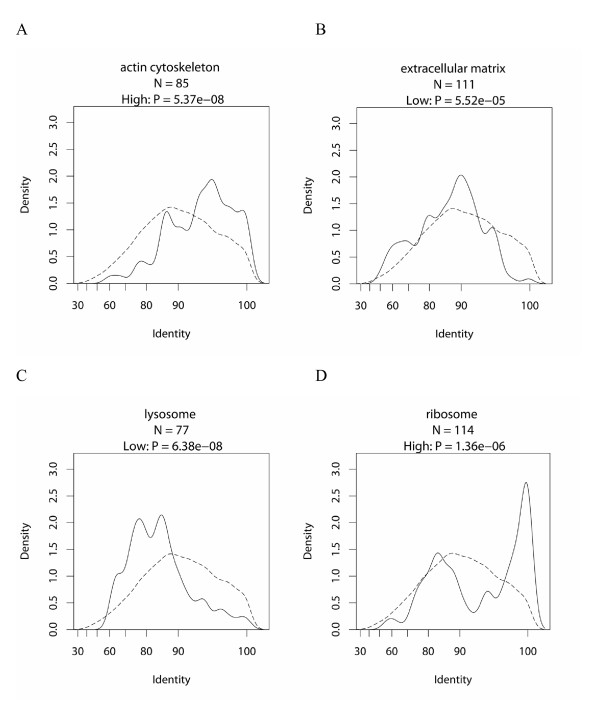
**Distribution of percentage identities of human and mouse protein sequences**. For the high conservation tendency, actin cytoskeleton (A) and ribosome (D), for the low conservation tendency, extracellular matrix (B) and lysosome (C). For each of A-D, the solid line shows the distribution of the identities for genes with the specific GO term, and the dashed line shows the distribution for the control gene set (see Materials and Methods for details).

The protein conservation tendencies were examined in a similar manner to those of the promoter conservation, using protein sequences obtained from the RefSeq database. Since the alignment score largely depends on the protein length, we used the percentage identity for protein sequences, instead of the alignment scores. GO terms showing high protein conservation are listed in Table 3, and those with low conservation are in Table 4 (only terms with a P-value < 0.01; for the complete list of results, see Additional file 5). Figure 3 shows the distributions of conservation levels for several GO terms with significant tendencies (all of the distributions for the GO terms in Table 3 and 4 are shown in Additional file 6). When we tried global alignment, we obtained quite similar tendencies (data not shown), which is reasonable, given that the coverages of the local alignments were mostly over 95% (data not shown).

**Table 3:** GO categories with high protein conservation. Terms of biological process are labeled as P, cellular component as C, molecular function as F.

**GO term**	**Number of genes**	**P-value**
F:GTPase activity	88	0
F:GTP binding	160	0
P:intracellular transport	350	0
P:small GTPase mediated signal transduction	126	1.11E-16
F:RNA binding	290	1.33E-15
C:cytosol	171	3.70E-11
P:RNA processing	198	3.25E-10
C:Golgi apparatus	216	5.63E-10
P:intracellular signaling cascade	431	2.57E-09
C:spliceosome complex	37	6.46E-09
P:transcription	640	1.76E-08
P:regulation of transcription	602	2.02E-08
F:ATP binding	520	2.85E-08
C:actin cytoskeleton	85	5.37E-08
P:vesicle-mediated transport	190	7.02E-08
P:cytoskeleton organization and biogenesis	155	9.26E-08
F:cytoskeletal protein binding	137	1.44E-07
P:secretory pathway	102	7.91E-07
C:nucleoplasm	107	1.22E-06
C:ribosome	114	1.36E-06
P:protein biosynthesis	283	1.56E-06
P:ubiquitin cycle	235	2.86E-06
F:ion channel activity	98	7.08E-05
P:protein amino acid phosphorylation	213	0.000101
F:ATPase activity	130	0.000143
C:endomembrane system	163	0.000154
F:protein kinase activity	220	0.000178
P:nervous system development	154	0.000293
F:transcription factor activity	263	0.000465
C:microtubule cytoskeleton	115	0.000595
C:vesicle	86	0.000732
F:structural molecule activity	307	0.000801
F:structural constituent of ribosome	130	0.000843
F:ubiquitin-protein ligase activity	144	0.001969
C:organelle membrane	242	0.004122
P:cell cycle	340	0.006445

**Table 4:** GO categories with low protein conservation. Terms of biological process are labeled as P, cellular component as C, molecular function as F.

**GO term**	**Number of genes**	**P-value**
P:response to biotic stimulus	318	4.08E-49
P:immune response	270	1.16E-44
C:extracellular space	179	3.49E-37
P:response to stress	446	5.05E-26
F:oxidoreductase activity	309	2.35E-12
F:receptor activity	391	1.11E-11
F:receptor binding	221	2.15E-11
P:lipid metabolism	260	5.95E-11
P:electron transport	151	7.64E-10
C:lysosome	77	6.38E-08
F:peptidase activity	227	6.15E-07
P:cell proliferation	258	1.65E-06
P:cell adhesion	242	2.16E-06
C:mitochondrion	398	3.00E-05
P:proteolysis	259	4.53E-05
C:extracellular matrix	111	5.52E-05
C:peroxisome	49	8.02E-05
F:nuclease activity	60	8.50E-05
C:plasma membrane	608	0.000291
P:apoptosis	244	0.001373
P:carboxylic acid metabolism	225	0.002527
P:response to abiotic stimulus	148	0.004444
P:positive regulation of biological process	275	0.004599
P:response to chemical stimulus	129	0.004626
P:lipid biosynthesis	101	0.005576
P:cell-cell signaling	176	0.006021
P:sensory perception	111	0.008042

Table 3 includes well-known categories for high protein conservation: actins [4], ribosomal proteins, Ras-like GTPases [7] and RNA processing [34], and for low protein conservation, P:immune response [6]. By looking over Table 3, we realized that the categories are composed of a series of processes required for gene expression; from intracellular signaling cascade and regulation of transcription, to RNA processing, protein biosynthesis and intracellular transport. We also find C:cytosol and C:nucleoplasm, where the above-mentioned processes take place, and C:actin cytoskeleton, which is known to be involved in transcription [35]. On the other hand, in Table 4, the terms with low conservation are related to extracellular regions or cell surface (C:extracellular space, C:extracellular matrix, C:plasma membrane, F:receptor activity, F:receptor binding, P:cell-cell signaling or P:cell adhesion) or to membrane-bounded organelles (C:lysosome, C:mitochondrion or C:peroxisome). Other terms, such as F:oxidoreductase activity, F:peptidase activity, F:nuclease activity, P:electron tansport and P:proteolysis, correspond to the functions of these cellular components.

### Relationship between promoter conservation and protein conservation

To examine the relationship between promoter conservation and protein conservation, we calculated the correlation coefficient of promoter conservation (raw alignment score obtained by **water**) and protein conservation (percentage identity obtained by **water**). This correlation was very weak (the Kendall's rank correlation is 0.193, see Additional file 7 for the scatter plot), suggesting that the promoter and protein sequences are under different types of selective pressure. We further investigated the relationship between protein and promoter conservation in detail, by decomposing it based on GO categories. From Tables 1, 2, 3 and 4, the terms that have significant conservation tendencies for both protein sequences and promoter sequences were extracted and compiled as a 2 by 2 cross table (Table 5). Although this table was basically made by the GO annotations of human genes, the results of the same analysis based on mouse annotations are superimposed, as both analyses were consistent. P:cell-cell signaling was the only exceptional case, showing low protein conservation based on human annotation and high protein conservation on mouse annotation. An examination of the contents of the two gene sets revealed that the observed difference seems to be derived from the different GO annotation status between human and mouse. Specifically, 151 genes out of 176 are annotated as P:cell-cell signaling only in human, and these genes seems to contribute to the low protein conservation tendency (see Additional file 8). Since human annotations are more abundant, we made the tables with human annotations, and added marks for mouse annotations.

**Table 5:** Summary of GO categories that show significant conservation tendencies for both protein and promoter sequences.

**Promoter conservation**			
	**High**	F:receptor binding (221) *	P:regulation of transcription (602) *
		P:cell-cell signaling (176) *	F:transcription factor activity (263) *
		C:extracellular matrix (111) *	P:transcription (640) *
		P:cell adhesion (242)	P:nervous system development (154) *
		C:plasma membrane (608)	F:cytoskeletal protein binding (137) *
			F:ion channel activity (98) *
			C:actin cytoskeleton (85) *
			P:protein amino acid phosphorylation (213) *
			F:protein kinase activity (220) *
			P:intracellular signaling cascade (431) *
		
	**Low**	P:proteolysis (259) *	P:protein biosynthesis (283) *
		F:peptidase activity (227) *	F:structural constituent of ribosome (130) *
		P:immune response (270)	C:ribosome (114) *
		P:response to biotic stimulus (318)	
		F:nuclease activity (60) *	
		C:peroxisome (49) *	
		P:electron transport (151) *	
		P:carboxylic acid metabolism (225)	
		P:lipid metabolism (260) *	
		C:lysosome (77) *	
		F:oxidoreductase activity (309) *	
		C:mitochondrion (398) *	
		**Low**	**High**
		**Protein conservation**	

In each cell, the GO categories are ordered by promoter conservation. The number of genes for each term is shown in parentheses. GO annotations associated with human genes were used to make this table. '*' represent GO terms that show a significant tendency not only for the human annotation but also for the mouse annotation.

Table 5 illustrates the relationship between protein conservation and promoter conservation, on the functional category basis. GO terms in the upper right cell, which have high conservation for both protein and promoter sequences, are related to transcription regulation or intracellular signaling. In contrast, the membrane-bounded organelles engaged in metabolism are in the lower left cell, showing low conservation for both protein and promoter. Interestingly, several terms are in the upper left and lower right cell, indicating opposite characteristics for protein and promoter conservation. For example, although genes related to signaling events showed high promoter conservation, they do not always have high protein conservation, but can even have low protein conservation; P:cell-cell signaling shows low protein conservation, while F:regulation of transcription shows high protein conservation. An analogous situation can be seen in the case of genes with low promoter conservation; among metabolism-related terms, C:ribosome shows high protein conservation, while C:mitochondrion shows low protein conservation. These results illustrate that there seems to be a nonparallel component in protein and promoter sequence evolution.

### Protein and promoter conservation of ribosomal proteins

Unlike other categories, C:ribosome shows a bimodal distribution of protein conservation (Figure 3B); one is around 100% identity, and the other ranges from 70% to 90%. Consistently, several categories related to C:ribosome (P:protein biosynthesis and F:structural constituent of ribosome) also show bimodal distributions (Additional file 6). This result could be due to different evolutionary rates between cytoplasmic and mitochondrial ribosomal protein [36]. Therefore, we checked the annotations for the genes in the C:ribosome category, using the NCBI RefSeq database [37]. In fact, the peak with high protein conservation is substantially composed of cytoplasmic ribosomal proteins, while the peak with lower protein conservation mainly comprises nuclear-encoded mitochondrial ribosomal proteins (Additional file 9). Notably, the general protein conservation tendency described in previous sections holds here: proteins in the cytosol show high protein conservation, while proteins in membrane-bounded organelles, such as mitochondria, have low protein conservation.

**Figure 4:**
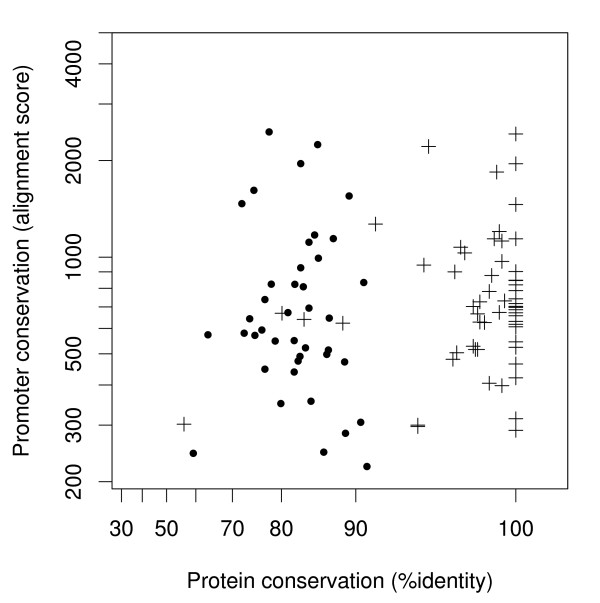
**Scatter plot of protein conservation and promoter conservation for two subsets of ribosomal proteins**. Crosses represent cytoplasmic ribosomal proteins (58 genes). Dots represent mitochondrial ribosomal proteins (41 genes). The conspicuous outlier corresponding to (56, 302) does not seem to be an actual ribosomal protein, and might have been erroneously annotated by an electronic procedure (see Additional file 9).

Besides the protein conservation, we examined the promoter conservation tendency for the two subsets of the C:ribosome category, cytoplasmic and mitochondrial ribosomal proteins. In contrast to the protein conservation, we could not observe a significant difference in the conservation levels between these two subgroups (P-value = 0.34 by Wilcoxon rank sum test; see Additional file 10 for details of the distributions). The plot of promoter conservation levels against protein conservation is shown in Figure 4. Apparently, the protein conservation is drastically different between cytoplasmic and mitochondrial ribosomal proteins, whereas the distribution of promoter conservation is quite similar. This result underscores the decoupled property of protein and promoter sequence evolution.

## Discussion

When we conducted a comprehensive comparison of promoter sequences for human and mouse orthologous genes, we noted that the promoter pairs of orthologous genes contained non-orthologous promoters. The source of these non-orthologous promoters could be the potential false pairings in the orthologous table. Another possible reason is the presence of alternative promoters [38,39], which can result in the failure to select the corresponding TSSs between human and mouse. The other possible cause is the existence of species-specific promoters; for example, our group recently reported that there are human promoter sequences whose counterparts are completely missing in the mouse genomic sequences [40]. Nevertheless, despite these problems that may cause mis-pairing of non-orthologous promoters, as much as 82% of the promoter pairs were shown to be evolutionally related in the data set. Although the dynamic aspects of TSSs, such as TSS diversification ad TSS turn over, have been highlighted recently [38,39,41,42], our results show that the representative TSS for each gene has been generally sustained during the evolution of the human and mouse lineages.

We focused on gene pairs with promoters that appeared to be truly evolutionally related, and examined the relationship between promoter conservation and gene function. We found that the terms with high promoter conservation are related to signaling events inside as well as outside of the cell. Considering that the promoter conservation levels reflect the regulatory information contained in the sequence, the results suggest that these genes require more regulatory information embedded in the promoter. It is reasonable to suppose that more regulatory information enables more sophisticated changes of expression levels, thereby allowing these proteins to work effectively as signaling molecules. On the other hand, genes involved in metabolism, which showed low promoter conservation, may require relatively less regulatory information in their promoter sequences. Consistently, a recent study revealed that housekeeping genes tend to show reduced upstream sequence conservation [43]. Specifically, in relation to ribosomal proteins, Perry et al. [44] pointed out that most of their promoters contain transposable elements, resulting in a low conservation. The reduced regulatory information in the promoters of ribosomal proteins might be compensated by the translational regulation mechanism directed by the 5' terminal oligopyrimidine sequence in their mRNAs [45].

Related discussions on regulatory sequence conservation have been made for specific categories of genes. Iwama and Gojobori [11] found that transcription factor genes, particularly those related to developmental processes, show high upstream sequence conservation, suggesting that these genes form highly connected regulatory networks. Lee et al. [12] reported that genes involved in adaptive processes tend to have highly conserved upstream regions in mammalian genomes, and also suggested the complex combinatorial circuitry of their transcriptional regulation. There have been other approaches based on whole genome comparisons, where highly conserved non-coding regions were found to be associated with developmental genes [34,46,47]. However, as Lee et al. suggested [12], most of these regions are far from genes and have little overlap with promoter regions. Thus, it seems that these regions are conserved independently from the promoter regions.

The conserved elements in the promoter may be either very short, or spread over a much longer region than the 1,200 bases. In both cases, our measures will report poor conservation when there is just a right amount of it. The local alignment score we used to measure promoter conservation can be roughly considered as a combination of identity and alignment length. Identity reflects the rates of substitutions and indels, and length reflects larger insertions, such as transposon insertions. When we examined the promoter conservation tendency for each GO term, by using alignment length or percentage identity as a measure of conservation, the tendencies were consistent with each other (Additional file 11). Thus, the evolutionary pressures of each functional category on alignment length and identity work in the same direction.

When we investigated the relationship between protein conservation and promoter conservation in mammals, we observed a very weak correlation between them. This suggests that substantial portions of the evolutionary changes of promoter and protein sequences are under different types of selective pressures. This observation is consistent with the nematode [20] and yeast [48] cases, and thus the very weak correlation between protein and promoter conservation might be universal from unicellular organisms to higher vertebrates.

In order to understand the relationship of protein and promoter sequence conservation in terms of gene functions, we examined it by a decomposition based on GO categories. When we dissected not only promoter conservation but also protein conservation, different trends were observed for proteins and promoters. As for proteins, high conservations were observed for terms related to a wide range of gene expression processes that occur in the cytosol and the nucleoplasm, while low conservations were observed for terms related to extracellular regions, cell surface and membrane-bounded organelles (such as mitochondrion, peroxisome and lysosome). Although the results for the membrane-bounded organelles seem surprising, considering that they often carry out basic, conserved metabolic process, they can also be considered as being topologically "outside" of the cell, given that they are on the opposite side of the membrane from the cytosol. The problem of the determinant of the protein evolutionary rate [49,50] needs to be solved to fully clarify the phenomenon. Nevertheless, our observation provides the trends of the protein sequence evolution in terms of functional categories. Comparing these trends with those of promoters, we found that these two kinds of trends are nonparallel: protein conservation depends on whether they are on the cytosolic side or not, while promoter conservation seems to depend on whether the gene is related to signaling or metabolism. Specifically, cytoplasmic ribosomal proteins, which exist in the cytosol and are engaged in metabolism, have high protein conservation in spite of low promoter conservation. On the other hand, cell-cell signaling genes, which act outside or at the surface of the cell to convey signals, show low protein conservation in spite of high promoter conservation. These terms may provide evidence that decoupled properties exist between protein and promoter sequence evolution.

## Conclusion

In this study, we examined the relationship between protein conservation and promoter conservation in detail, by decomposing it based on functional categories. Our results show the relation of gene function to protein conservation and promoter conservation, and revealed that there seem to be nonparallel components between protein and promoter sequence evolution. We believe that this study will provide a basis to understand the evolution of mammalian genes and their regulation. Further efforts are now being made to construct reliable promoter sequences based on full-length cDNAs. Future analyses of multiple species will clarify the evolutionary mechanisms of the coding and regulatory sequences more precisely.

## Methods

### Sequence comparison

From DBTSS, we obtained human and mouse orthologous gene pairs with experimentally validated TSS information. The definition of an orthologous relationship is based on HomoloGene [51]. One-to-multi orthologous relationships were removed, resulting in 8,429 one-to-one orthologous gene pairs. Since the TSSs for a given gene are not fixed but vary on the chromosome, a representative TSS was defined for each gene, as described in Yamashita et al. [27]. Based on the positions of representative TSSs, sequences from -1000 to +200 were defined as putative promoter sequences. Promoters of orthologous gene pairs were aligned by the local alignment program **water **from the EMBOSS package [33]. In addition, promoter pairs to be used as a negative control were created by shuffling the original pairings, and were aligned similarly. The protein sequences of orthologous gene pairs were obtained from the NCBI reference sequence (RefSeq) database [37]. They were also aligned with **water**. For additional analyses by global alignments, **needle **from the EMBOSS package was used. Furthermore, we confirmed the results after eliminating coding sequences contained in promoter sequences, as follows. The coding sequences downstream of the TSSs were removed by restricting the promoter sequences from -1000 to -1 of the TSSs. In addition, since 16% of the shortened sequences (1,101 out of 6,901) still contained coding sequences, we used the other 5,800 sequences for the additional analyses.

To display the distributions of the alignment scores, they were transformed by common logarithmic transformation, and then the densities were estimated by R with the Gaussian kernel and a band width of 0.5. For protein sequences, protein diversity, instead of identity, was subjected to the logarithmic transformation. In addition, to avoid zero before the logarithmic transformation, a small number was added. Thus, 105 - *identity *was subjected to the logarithmic transformation. This transformation is similar to that described in a previous study on protein evolutionary rates [49].

### Annotations of genes

Annotations of genes were based on the gene ontology (GO) [52]. The GO annotations for the human and mouse genes were obtained from the gene2go file at NCBI [53]. In this study, to summarize the attributes of the genes, we developed a slimmed-down version of the GO vocabulary (GO slim), as follows. A set of high level terms was selected to cover most aspects of each of the three ontologies (52 terms for biological process, 22 terms for cellular component and 26 terms for molecular function; for the complete list of selected GO terms, see Additional file 2). Basically, GO terms containing over 100 genes were selected, although well-known cellular components with smaller number of genes, such as C:lysosome and C:peroxisome, were also included. Overly general terms, such as C:cell, P:physiological process and F:binding, were removed, because their biological interpretation seems uninformative. Each GO term was mapped to the GO slim terms using map2slim.pl from the go-perl package [54]. Note that several GO slim terms can be assigned to a single gene; that is, the GO slim terms are not mutually exclusive. In the other sections of the paper, the GO slim terms are referred to as "GO term" for short.

### Significance test for the extent of conservation

We tested whether the alignment scores (or percentage identities) of a set of genes associated with a given GO term are significantly high or low by a Wilcoxon rank sum test. It should be noted that the genes used as a control group of a term are those that are not associated with the term, but with other terms. For example, in the case of 'transcription' of biological process, 640 genes are associated with the term among 6,901 genes. Of the 6,261 genes that are not associated with 'transcription', 2,116 genes are missing terms of biological processes. Since these "uncharacterized" genes had low sequence conservation tendencies, we eliminated them from the control gene set. The resulting control set in the case of 'transcription' is thus composed of 4,145 genes.

## Authors' contributions

HC carried out the analysis and drafted the manuscript. RY, KK and KN were involved in the study design. KN coordinated the research. All authors read and approved the final manuscript.

## Supplementary Material

Additional file 1 Estimated distributions of orthologous and non-orthologous promoter pairs contained in the dataset. Click here for file

Additional file 2 Complete list of 100 GO terms selected for this analysis. Click here for file

Additional file 3 Complete list of the results of significance tests for promoter conservation of each category. Click here for file

Additional file 4 **Distribution of alignment scores of human and mouse promoters for GO categories with significant tendencies**. A. GO categories with high protein conservation, B. GO categories with low protein conservation. Click here for file

Additional file 5 Complete list of the results of significance tests for protein conservation of each category. Click here for file

Additional file 6 **Distribution of percentage identities of human and mouse protein sequences for GO categories with significant tendencies**. A. GO categories with high protein conservation, B. GO categories with low protein conservation. Click here for file

Additional file 7 Scatter plot of protein conservation and promoter conservation for human and mouse orthologous genes. Click here for file

Additional file 8 Protein conservation of human and mouse 'cell-cell signaling' genes. Click here for file

Additional file 9 **Protein conservations and RefSeq annotations for 'ribosome' category**. Genes are sorted by the percentage identity. Click here for file

Additional file 10 **Promoter conservations and RefSeq annotations for 'ribosome' category**. Genes are sorted by the alignment score. Click here for file

Additional file 11 Promoter conservation tendency for each GO category based on alignment length and percentage identity. Click here for file
