# Supplementary material for: Weak correlation between sequence conservation in promoter regions and in protein-coding regions of human-mouse orthologous gene pairs
Source: BMC Genomics. 2008 Apr 2;9:152. doi: 10.1186/1471-2164-9-152 (PMC2335122; doi:10.1186/1471-2164-9-152)
Supplement: Additional file 1 — Estimated distributions of orthologous and non-orthologous promoter pairs contained in the dataset. [file 1471-2164-9-152-S1.pdf]

Additional file 1

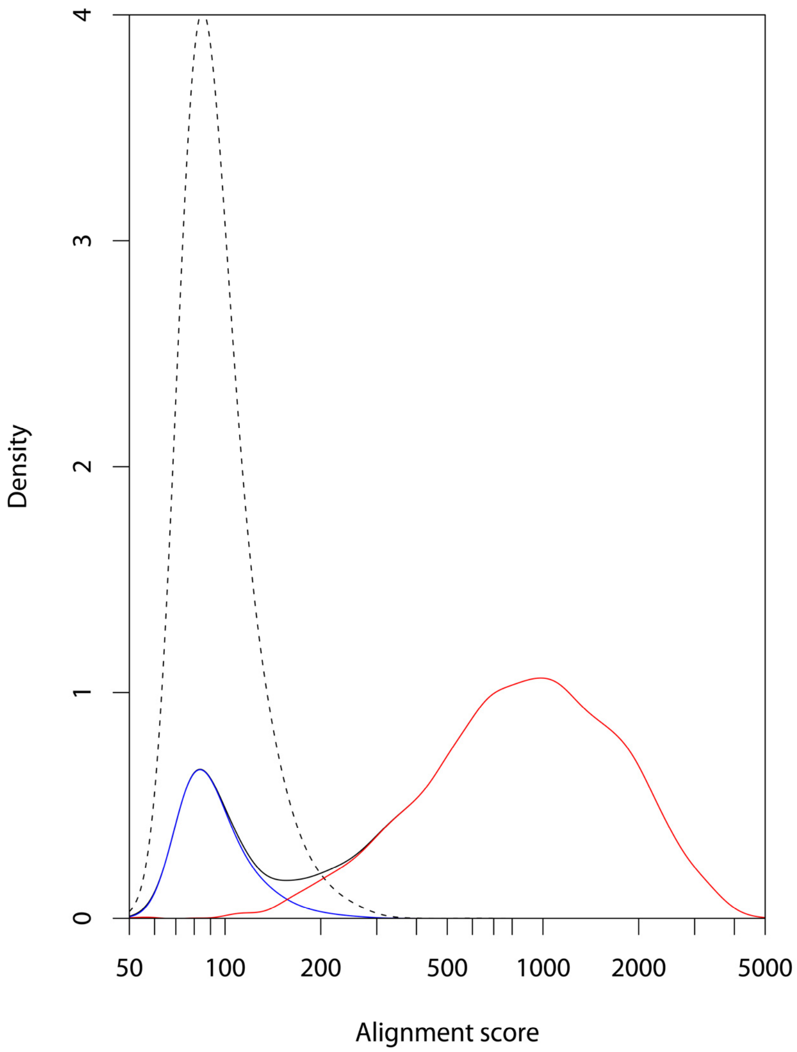

- - - - Negative control

Estimated proportion of non-orthologous promoters

Estimated proportion of orthologous promoters
