# Supplementary material for: Weak correlation between sequence conservation in promoter regions and in protein-coding regions of human-mouse orthologous gene pairs
Source: BMC Genomics. 2008 Apr 2;9:152. doi: 10.1186/1471-2164-9-152 (PMC2335122; doi:10.1186/1471-2164-9-152)
Supplement: Additional file 2 — Complete list of 100 GO terms selected for this analysis. [file 1471-2164-9-152-S2.pdf]

Additional file 2

GO ID	ontology	GO term
GO:0000902	biological process	cellular morphogenesis
GO:0005975	biological process	carbohydrate metabolism
GO:0006066	biological process	alcohol metabolism
GO:0006118	biological process	electron transport
GO:0006259	biological process	DNA metabolism
GO:0006350	biological process	transcription
GO:0006396	biological process	RNA processing
GO:0006412	biological process	protein biosynthesis
GO:0006457	biological process	protein folding
GO:0006461	biological process	protein complex assembly
GO:0006468	biological process	protein amino acid phosphorylation
GO:0006508	biological process	proteolysis
GO:0006512	biological process	ubiquitin cycle
GO:0006520	biological process	amino acid metabolism
GO:0006629	biological process	lipid metabolism
GO:0006811	biological process	ion transport
GO:0006915	biological process	apoptosis
GO:0006928	biological process	cell motility
GO:0006950	biological process	response to stress
GO:0006955	biological process	immune response
GO:0007010	biological process	cytoskeleton organization and biogenesis
GO:0007049	biological process	cell cycle
GO:0007155	biological process	cell adhesion
GO:0007165	biological process	signal transduction
GO:0007166	biological process	cell surface receptor linked signal transduction
GO:0007186	biological process	G-protein coupled receptor protein signaling pathway
GO:0007242	biological process	intracellular signaling cascade
GO:0007243	biological process	protein kinase cascade
GO:0007264	biological process	small GTPase mediated signal transduction
GO:0007267	biological process	cell-cell signaling
GO:0007275	biological process	development
GO:0007399	biological process	nervous system development
GO:0007600	biological process	sensory perception
GO:0008283	biological process	cell proliferation
GO:0008610	biological process	lipid biosynthesis
GO:0009056	biological process	catabolism
GO:0009607	biological process	response to biotic stimulus
GO:0009628	biological process	response to abiotic stimulus
GO:0009653	biological process	morphogenesis
GO:0009892	biological process	negative regulation of metabolism
GO:0016192	biological process	vesicle-mediated transport
GO:0019752	biological process	carboxylic acid metabolism
GO:0030154	biological process	cell differentiation
GO:0042221	biological process	response to chemical stimulus
GO:0045045	biological process	secretory pathway
GO:0045449	biological process	regulation of transcription
GO:0046907	biological process	intracellular transport
GO:0048513	biological process	organ development
GO:0048518	biological process	positive regulation of biological process
GO:0048519	biological process	negative regulation of biological process
GO:0051186	biological process	cofactor metabolism
GO:0051276	biological process	chromosome organization and biogenesis
GO:0000151	cellular component	ubiquitin ligase complex
GO:0005615	cellular component	extracellular space
GO:0005654	cellular component	nucleoplasm
GO:0005681	cellular component	spliceosome complex
GO:0005694	cellular component	chromosome
GO:0005730	cellular component	nucleolus
GO:0005739	cellular component	mitochondrion
GO:0005764	cellular component	lysosome
GO:0005768	cellular component	endosome
GO:0005777	cellular component	peroxisome
GO:0005783	cellular component	endoplasmic reticulum

GO:0005794	cellular component	Golgi apparatus
GO:0005829	cellular component	cytosol
GO:0005840	cellular component	ribosome
GO:0005886	cellular component	plasma membrane
GO:0012505	cellular component	endomembrane system
GO:0015629	cellular component	actin cytoskeleton
GO:0015630	cellular component	microtubule cytoskeleton
GO:0031012	cellular component	extracellular matrix
GO:0031090	cellular component	organelle membrane
GO:0031967	cellular component	organelle envelope
GO:0031982	cellular component	vesicle
GO:0000287	molecular function	magnesium ion binding
GO:0003677	molecular function	DNA binding
GO:0003700	molecular function	transcription factor activity
GO:0003723	molecular function	RNA binding
GO:0003735	molecular function	structural constituent of ribosome
GO:0003924	molecular function	GTPase activity
GO:0004518	molecular function	nuclease activity
GO:0004672	molecular function	protein kinase activity
GO:0004842	molecular function	ubiquitin-protein ligase activity
GO:0004872	molecular function	receptor activity
GO:0005102	molecular function	receptor binding
GO:0005198	molecular function	structural molecule activity
GO:0005216	molecular function	ion channel activity
GO:0005386	molecular function	carrier activity
GO:0005506	molecular function	iron ion binding
GO:0005509	molecular function	calcium ion binding
GO:0005524	molecular function	ATP binding
GO:0005525	molecular function	GTP binding
GO:0008092	molecular function	cytoskeletal protein binding
GO:0008233	molecular function	peptidase activity
GO:0008270	molecular function	zinc ion binding
GO:0015075	molecular function	ion transporter activity
GO:0016491	molecular function	oxidoreductase activity
GO:0016887	molecular function	ATPase activity
GO:0030234	molecular function	enzyme regulator activity
GO:0042578	molecular function	phosphoric ester hydrolase activity
