# Supplementary material for: Weak correlation between sequence conservation in promoter regions and in protein-coding regions of human-mouse orthologous gene pairs
Source: BMC Genomics. 2008 Apr 2;9:152. doi: 10.1186/1471-2164-9-152 (PMC2335122; doi:10.1186/1471-2164-9-152)
Supplement: Additional file 3 — Complete list of the results of significance tests for promoter conservation of each category. [file 1471-2164-9-152-S3.pdf]

Additional file 3

| GO term | number of samples | number of control | P-value for high conservation | P-value for low conservation |
|---|---|---|---|---|
| P:development | 649 | 4136 | 0 | 1.000000 |
| P:regulation of transcription | 602 | 4183 | 1.67E-15 | 1.000000 |
| F:transcription factor activity | 263 | 4760 | 3.44E-15 | 1.000000 |
| P:transcription | 640 | 4145 | 4.11E-14 | 1.000000 |
| P:nervous system development | 154 | 4631 | 1.99E-10 | 1.000000 |
| P:organ development | 213 | 4572 | 2.30E-10 | 1.000000 |
| P:signal transduction | 994 | 3791 | 5.19E-10 | 1.000000 |
| F:DNA binding | 628 | 4395 | 3.19E-08 | 1.000000 |
| P:morphogenesis | 212 | 4573 | 9.78E-08 | 1.000000 |
| P:cell surface receptor linked signal transduction | 363 | 4422 | 2.23E-06 | 0.999998 |
| P:negative regulation of metabolism | 107 | 4678 | 1.02E-05 | 0.999990 |
| F:receptor binding | 221 | 4802 | 1.90E-05 | 0.999981 |
| P:cell-cell signaling | 176 | 4609 | 2.27E-05 | 0.999977 |
| F:cytoskeletal protein binding | 137 | 4886 | 4.97E-05 | 0.999950 |
| P:negative regulation of biological process | 327 | 4458 | 6.87E-05 | 0.999931 |
| F:ion channel activity | 98 | 4925 | 9.87E-05 | 0.999901 |
| C:extracellular matrix | 111 | 4401 | 0.000119 | 0.999881 |
| C:actin cytoskeleton | 85 | 4427 | 0.000164 | 0.999837 |
| P:cell differentiation | 173 | 4612 | 0.000179 | 0.999821 |
| P:cell adhesion | 242 | 4543 | 0.000182 | 0.999818 |
| P:cellular morphogenesis | 111 | 4674 | 0.000607 | 0.999393 |
| F:ion transporter activity | 237 | 4786 | 0.001493 | 0.998508 |
| P:protein amino acid phosphorylation | 213 | 4572 | 0.001593 | 0.998407 |
| P:ion transport | 239 | 4546 | 0.001825 | 0.998176 |
| F:protein kinase activity | 220 | 4803 | 0.002033 | 0.997967 |
| P:intracellular signaling cascade | 431 | 4354 | 0.006872 | 0.993129 |
| P:chromosome organization and biogenesis | 105 | 4680 | 0.007832 | 0.992170 |
| C:plasma membrane | 608 | 3904 | 0.008026 | 0.991975 |
| F:GTPase activity | 88 | 4935 | 0.011437 | 0.988566 |
| P:cytoskeleton organization and biogenesis | 155 | 4630 | 0.011868 | 0.988133 |
| P:small GTPase mediated signal transduction | 126 | 4659 | 0.012373 | 0.987629 |
| P:cell proliferation | 258 | 4527 | 0.016427 | 0.983575 |
| F:GTP binding | 160 | 4863 | 0.026124 | 0.973879 |
| F:calcium ion binding | 280 | 4743 | 0.058031 | 0.941974 |
| P:cell motility | 105 | 4680 | 0.061020 | 0.938989 |
| F:receptor activity | 391 | 4632 | 0.078737 | 0.921269 |
| F:structural molecule activity | 307 | 4716 | 0.079056 | 0.920950 |
| P:G-protein coupled receptor protein signaling pathway | 153 | 4632 | 0.108505 | 0.891506 |
| F:phosphoric ester hydrolase activity | 113 | 4910 | 0.138352 | 0.861663 |
| F:enzyme regulator activity | 238 | 4785 | 0.148055 | 0.851956 |
| P:protein complex assembly | 122 | 4663 | 0.154119 | 0.845897 |
| P:vesicle-mediated transport | 190 | 4595 | 0.166697 | 0.833317 |
| P:protein kinase cascade | 118 | 4667 | 0.173566 | 0.826451 |
| C:nucleoplasm | 107 | 4405 | 0.198841 | 0.801180 |
| C:Golgi apparatus | 216 | 4296 | 0.202850 | 0.797165 |
| F:zinc ion binding | 600 | 4423 | 0.232496 | 0.767514 |
| F:carrier activity | 149 | 4874 | 0.239577 | 0.760441 |
| P:intracellular transport | 350 | 4435 | 0.240786 | 0.759226 |
| P:sensory perception | 111 | 4674 | 0.293922 | 0.706102 |
| P:cell cycle | 340 | 4445 | 0.297867 | 0.702148 |
| F:RNA binding | 290 | 4733 | 0.320681 | 0.679334 |
| P:positive regulation of biological process | 275 | 4510 | 0.345462 | 0.654555 |
| F:ATP binding | 520 | 4503 | 0.376693 | 0.623319 |
| F:ubiquitin-protein ligase activity | 144 | 4879 | 0.426143 | 0.573880 |
| P:DNA metabolism | 266 | 4519 | 0.454829 | 0.545189 |
| P:apoptosis | 244 | 4541 | 0.532993 | 0.467026 |
| C:microtubule cytoskeleton | 115 | 4397 | 0.540403 | 0.459626 |
| P:protein folding | 121 | 4664 | 0.586660 | 0.413366 |
| C:endoplasmic reticulum | 268 | 4244 | 0.591106 | 0.408913 |
| C:extracellular space | 179 | 4333 | 0.608820 | 0.391202 |
| P:response to chemical stimulus | 129 | 4656 | 0.645196 | 0.354828 |

| | | | | |
|---|---|---|---|---|
| F:ATPase activity | 130 | 4893 | 0.647215 | 0.352808 |
| P:ubiquitin cycle | 235 | 4550 | 0.654462 | 0.345556 |
| C:endomembrane system | 163 | 4349 | 0.667170 | 0.332852 |
| C:chromosome | 108 | 4404 | 0.692915 | 0.307111 |
| C:organelle envelope | 148 | 4364 | 0.696927 | 0.303096 |
| P:response to abiotic stimulus | 148 | 4637 | 0.720140 | 0.279881 |
| C:vesicle | 86 | 4426 | 0.734101 | 0.265927 |
| P:secretory pathway | 102 | 4683 | 0.777510 | 0.222512 |
| C:endosome | 33 | 4479 | 0.801091 | 0.198946 |
| C:ubiquitin ligase complex | 122 | 4390 | 0.815112 | 0.184907 |
| C:organelle membrane | 242 | 4270 | 0.832397 | 0.167616 |
| P:cofactor metabolism | 100 | 4685 | 0.901092 | 0.098920 |
| C:cytosol | 171 | 4341 | 0.924121 | 0.075887 |
| C:spliceosome complex | 37 | 4475 | 0.931290 | 0.068727 |
| F:magnesium ion binding | 135 | 4888 | 0.946019 | 0.053988 |
| P:alcohol metabolism | 133 | 4652 | 0.977205 | 0.022798 |
| P:lipid biosynthesis | 101 | 4684 | 0.978371 | 0.021633 |
| P:RNA processing | 198 | 4587 | 0.979718 | 0.020285 |
| P:response to stress | 446 | 4339 | 0.984169 | 0.015833 |
| P:proteolysis | 259 | 4526 | 0.993157 | 0.006844 |
| F:peptidase activity | 227 | 4796 | 0.994409 | 0.005592 |
| F:iron ion binding | 111 | 4912 | 0.994501 | 0.005500 |
| P:immune response | 270 | 4515 | 0.994563 | 0.005437 |
| C:nucleolus | 63 | 4449 | 0.995546 | 0.004455 |
| P:response to biotic stimulus | 318 | 4467 | 0.999107 | 0.000893 |
| F:nuclease activity | 60 | 4963 | 0.999228 | 0.000772 |
| P:protein biosynthesis | 283 | 4502 | 0.999370 | 0.000630 |
| C:peroxisome | 49 | 4463 | 0.999377 | 0.000623 |
| P:carbohydrate metabolism | 220 | 4565 | 0.999722 | 0.000278 |
| P:catabolism | 260 | 4525 | 0.999749 | 0.000251 |
| P:electron transport | 151 | 4634 | 0.999764 | 0.000236 |
| P:amino acid metabolism | 112 | 4673 | 0.999898 | 0.000102 |
| F:structural constituent of ribosome | 130 | 4893 | 0.999994 | 5.76E-06 |
| P:carboxylic acid metabolism | 225 | 4560 | 0.999996 | 4.43E-06 |
| P:lipid metabolism | 260 | 4525 | 0.999999 | 1.04E-06 |
| C:ribosome | 114 | 4398 | 0.999999 | 7.54E-07 |
| C:lysosome | 77 | 4435 | 1.000000 | 9.94E-08 |
| F:oxidoreductase activity | 309 | 4714 | 1.000000 | 2.07E-08 |
| C:mitochondrion | 398 | 4114 | 1.000000 | 5.31E-09 |
