# Supplementary material for: Weak correlation between sequence conservation in promoter regions and in protein-coding regions of human-mouse orthologous gene pairs
Source: BMC Genomics. 2008 Apr 2;9:152. doi: 10.1186/1471-2164-9-152 (PMC2335122; doi:10.1186/1471-2164-9-152)
Supplement: Additional file 4 — Distribution of alignment scores of human and mouse promoters for GO categories with significant tendencies. A. GO categories with high protein conservation, B. GO categories with low protein conservation. [file 1471-2164-9-152-S4.pdf]

Additional file 4

A

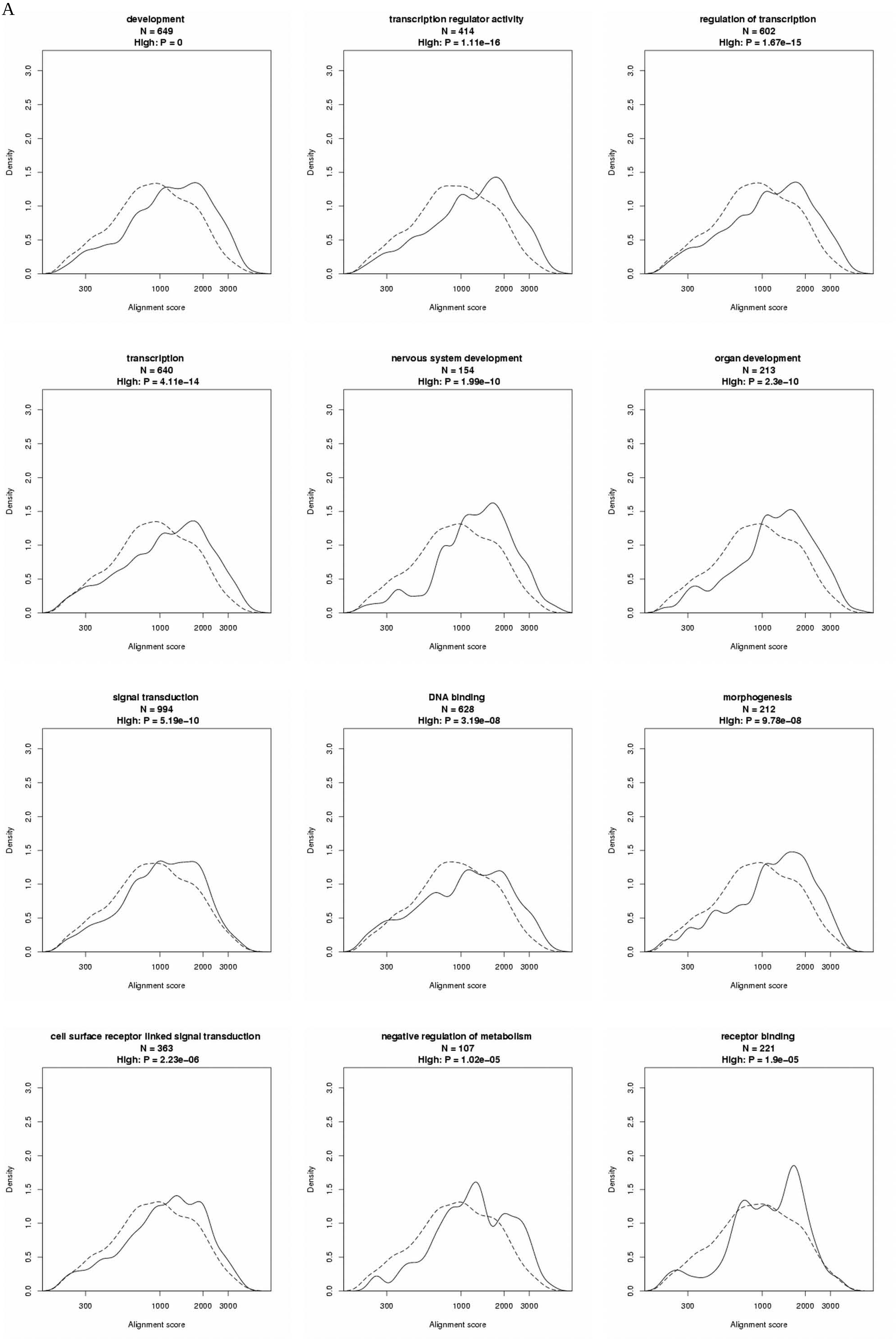

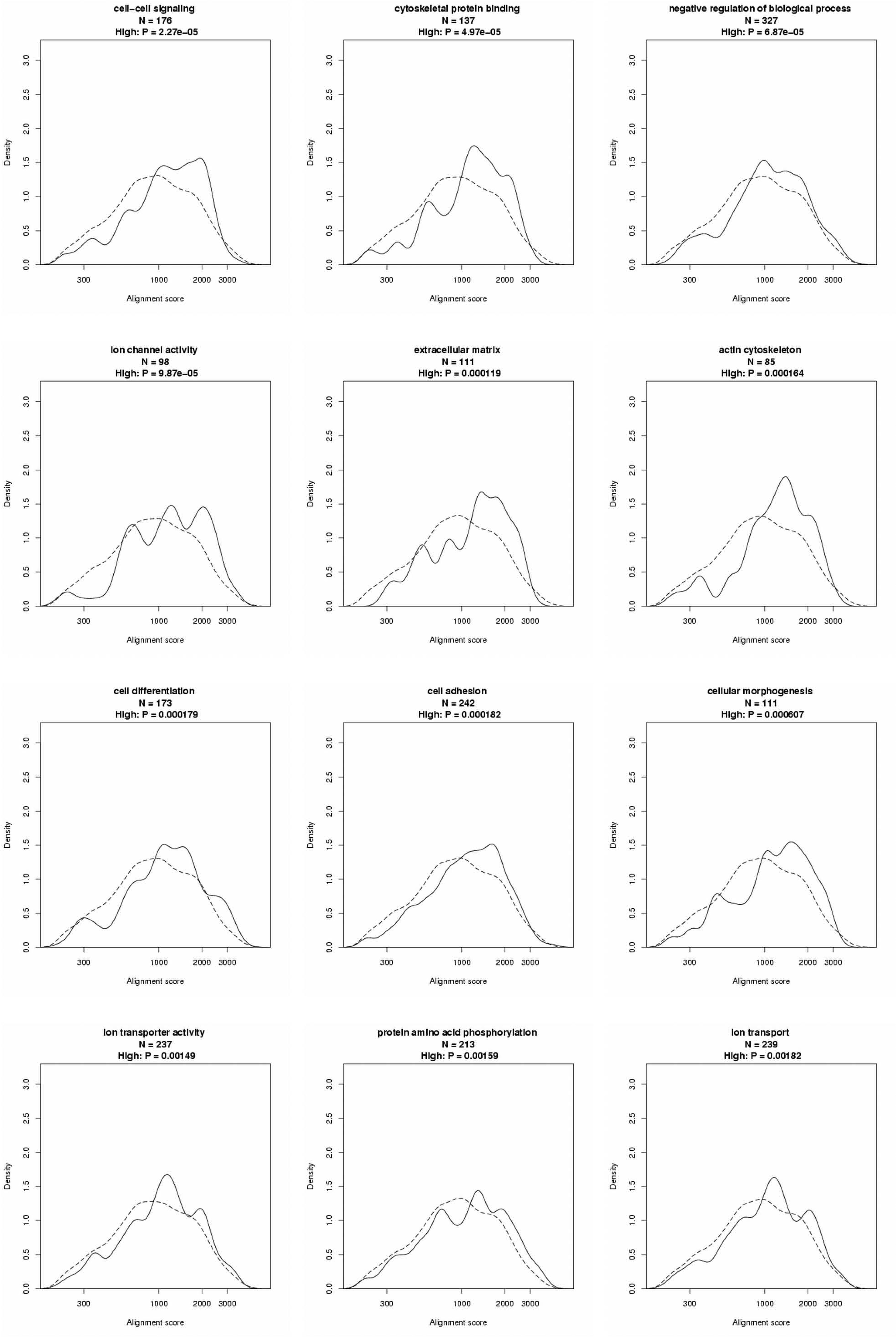
cell-cell signaling
N = 176
High: P = 2.27e-05
cytoskeletal protein binding
N = 137
High: P = 4.97e-05
negative regulation of biological process
N = 327
High: P = 6.87e-05
ion channel activity
N = 98
High: P = 9.87e-05
extracellular matrix
N = 111
High: P = 0.000119
actin cytoskeleton
N = 85
High: P = 0.000164
cell differentiation
N = 173
High: P = 0.000179
cell adhesion
N = 242
High: P = 0.000182
cellular morphogenesis
N = 111
High: P = 0.000607
ion transporter activity
N = 237
High: P = 0.00149
protein amino acid phosphorylation
N = 213
High: P = 0.00159
ion transport
N = 239
High: P = 0.00182
Density
0.0
0.5
1.0
1.5
2.0
2.5
3.0
Alignment score
300
1000
2000
3000

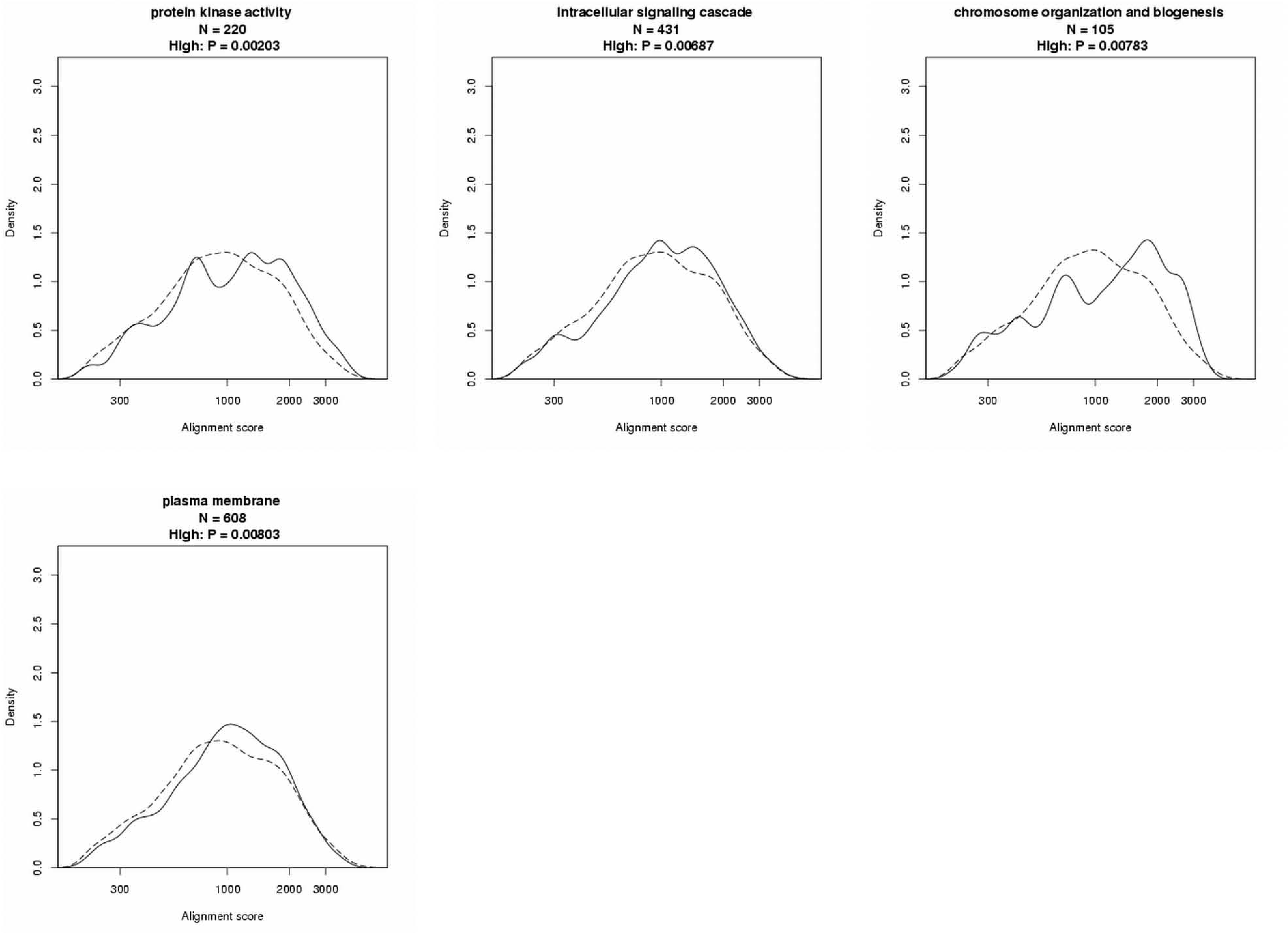
protein kinase activity
N = 220
High: P = 0.00203
intracellular signaling cascade
N = 431
High: P = 0.00687
chromosome organization and biogenesis
N = 105
High: P = 0.00783
plasma membrane
N = 608
High: P = 0.00803
Density
Alignment score
0.0
0.5
1.0
1.5
2.0
2.5
3.0
300
1000
2000
3000

B

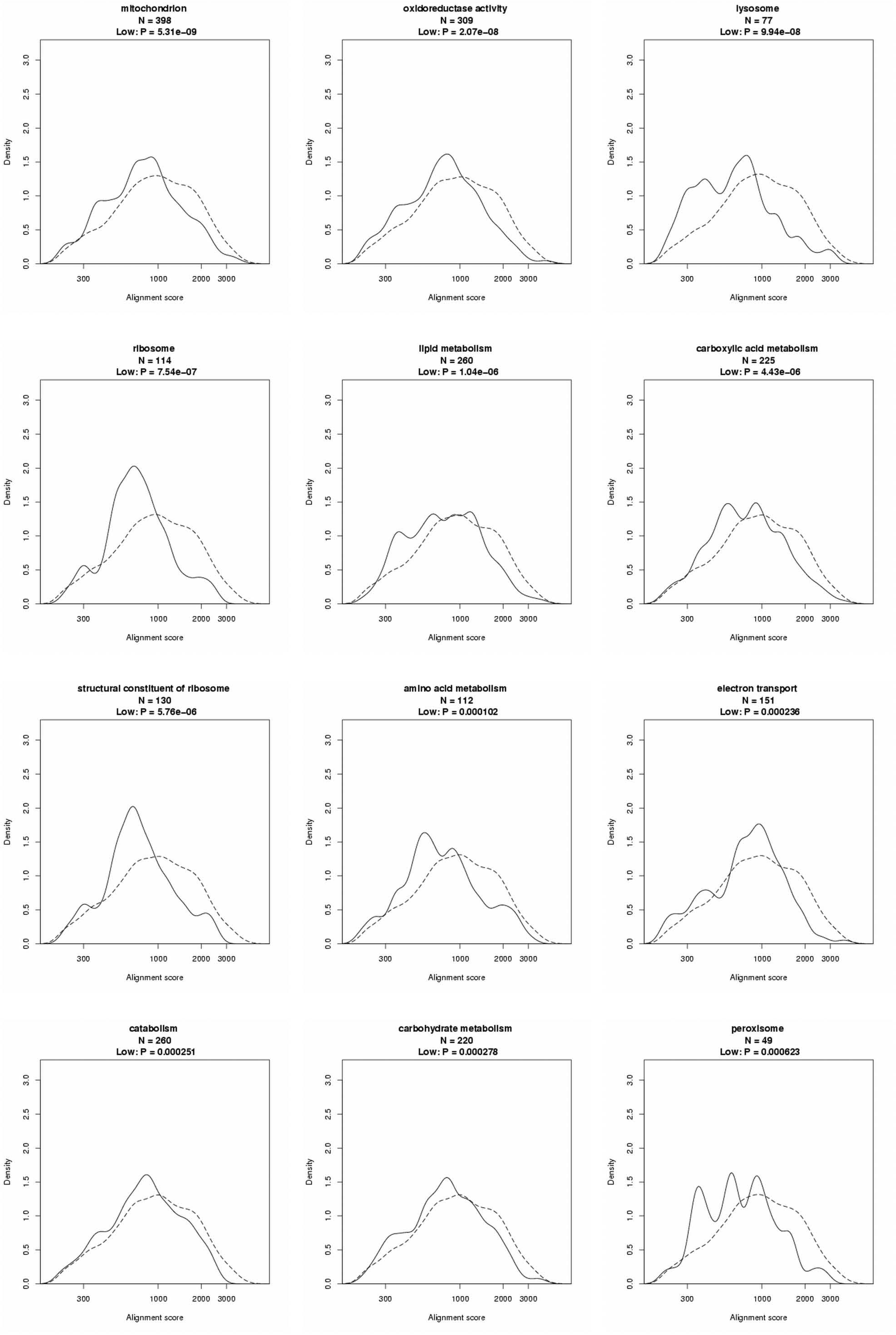

mitochondrion
N = 398
Low: P = 5.31e−09
oxidoreductase activity
N = 309
Low: P = 2.07e−08
lysosome
N = 77
Low: P = 9.94e−08
ribosome
N = 114
Low: P = 7.54e−07
lipid metabolism
N = 260
Low: P = 1.04e−06
carboxylic acid metabolism
N = 225
Low: P = 4.43e−06
structural constituent of ribosome
N = 130
Low: P = 5.76e−06
amino acid metabolism
N = 112
Low: P = 0.000102
electron transport
N = 151
Low: P = 0.000236
catabolism
N = 260
Low: P = 0.000251
carbohydrate metabolism
N = 220
Low: P = 0.000278
peroxisome
N = 49
Low: P = 0.000623
Density
0.0
0.5
1.0
1.5
2.0
2.5
3.0
300
1000
2000
3000
Alignment score

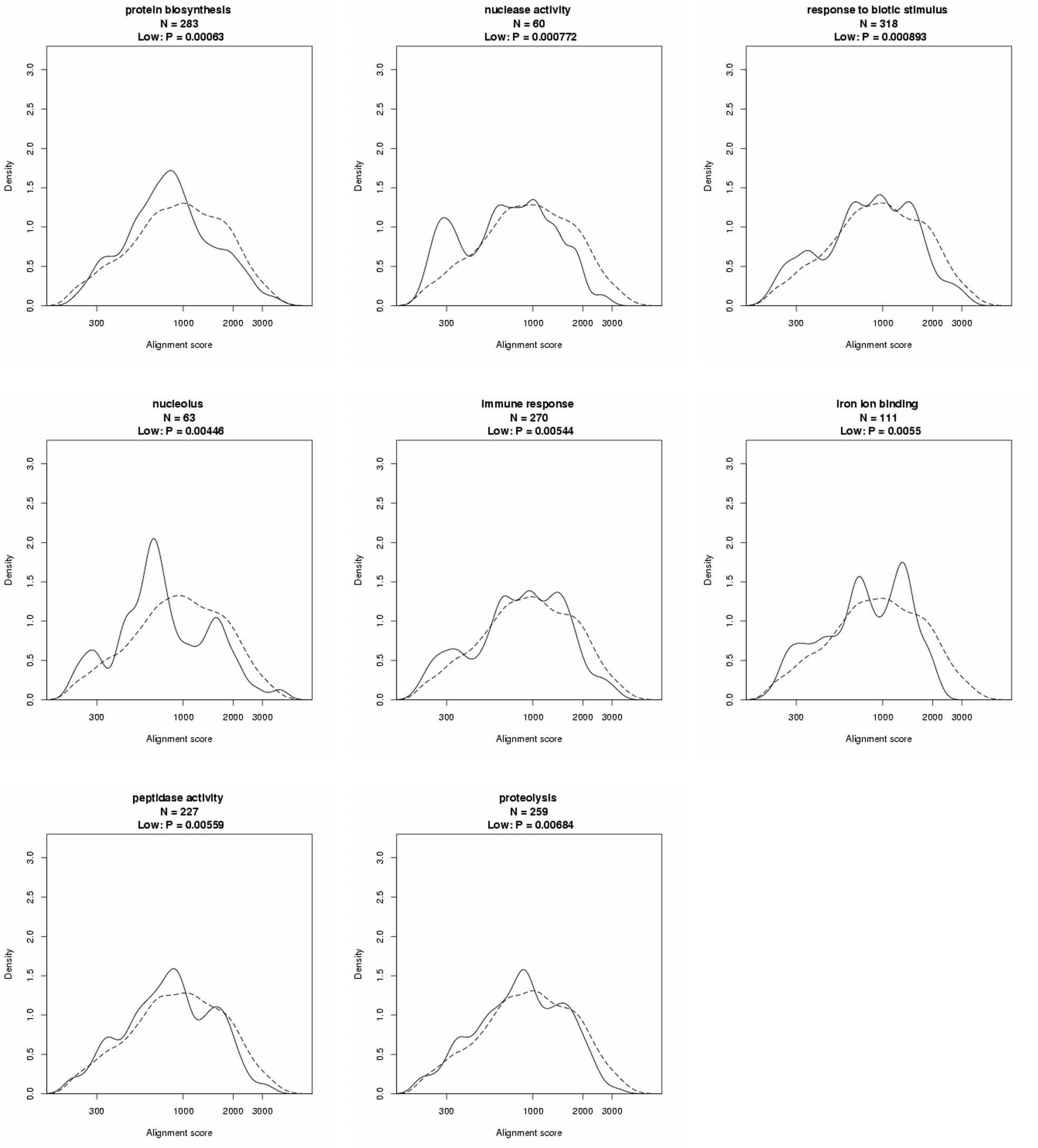
protein biosynthesis
N = 283
Low: P = 0.00063
nuclease activity
N = 60
Low: P = 0.000772
response to biotic stimulus
N = 318
Low: P = 0.000893
nucleolus
N = 63
Low: P = 0.00446
immune response
N = 270
Low: P = 0.00544
iron ion binding
N = 111
Low: P = 0.0055
peptidase activity
N = 227
Low: P = 0.00559
proteolysis
N = 259
Low: P = 0.00684
Density
0.0
0.5
1.0
1.5
2.0
2.5
3.0
300
1000
2000
3000
Alignment score
