# Supplementary material for: Weak correlation between sequence conservation in promoter regions and in protein-coding regions of human-mouse orthologous gene pairs
Source: BMC Genomics. 2008 Apr 2;9:152. doi: 10.1186/1471-2164-9-152 (PMC2335122; doi:10.1186/1471-2164-9-152)
Supplement: Additional file 5 — Complete list of the results of significance tests for protein conservation of each category. [file 1471-2164-9-152-S5.pdf]

Additional file 5

GO term	number of samples	number of control	P-value for high conservation	P-value for low conservation
F:GTPase activity	88	4935	0	1.000000
F:GTP binding	160	4863	0	1.000000
P:intracellular transport	350	4435	0	1.000000
P:small GTPase mediated signal transduction	126	4659	1.11E-16	1.000000
F:RNA binding	290	4733	1.33E-15	1.000000
C:cytosol	171	4341	3.70E-11	1.000000
P:RNA processing	198	4587	3.25E-10	1.000000
C:Golgi apparatus	216	4296	5.63E-10	1.000000
P:intracellular signaling cascade	431	4354	2.57E-09	1.000000
C:spliceosome complex	37	4475	6.46E-09	1.000000
P:transcription	640	4145	1.76E-08	1.000000
P:regulation of transcription	602	4183	2.02E-08	1.000000
F:ATP binding	520	4503	2.85E-08	1.000000
C:actin cytoskeleton	85	4427	5.37E-08	1.000000
P:vesicle-mediated transport	190	4595	7.02E-08	1.000000
P:cytoskeleton organization and biogenesis	155	4630	9.26E-08	1.000000
F:cytoskeletal protein binding	137	4886	1.44E-07	1.000000
P:secretory pathway	102	4683	7.91E-07	0.999999
C:nucleoplasm	107	4405	1.22E-06	0.999999
C:ribosome	114	4398	1.36E-06	0.999999
P:protein biosynthesis	283	4502	1.56E-06	0.999998
P:ubiquitin cycle	235	4550	2.86E-06	0.999997
F:ion channel activity	98	4925	7.08E-05	0.999929
P:protein amino acid phosphorylation	213	4572	0.000101	0.999899
F:ATPase activity	130	4893	0.000143	0.999857
C:endomembrane system	163	4349	0.000154	0.999846
F:protein kinase activity	220	4803	0.000178	0.999822
P:nervous system development	154	4631	0.000293	0.999707
F:transcription factor activity	263	4760	0.000465	0.999535
C:microtubule cytoskeleton	115	4397	0.000595	0.999405
C:vesicle	86	4426	0.000732	0.999268
F:structural molecule activity	307	4716	0.000801	0.999199
F:structural constituent of ribosome	130	4893	0.000843	0.999157
F:ubiquitin-protein ligase activity	144	4879	0.001969	0.998031
C:organelle membrane	242	4270	0.004122	0.995878
P:cell cycle	340	4445	0.006445	0.993555
F:ion transporter activity	237	4786	0.012295	0.987707
P:ion transport	239	4546	0.030566	0.969438
P:protein folding	121	4664	0.034044	0.965961
P:chromosome organization and biogenesis	105	4680	0.036151	0.963855
P:negative regulation of metabolism	107	4678	0.036606	0.963400
C:organelle envelope	148	4364	0.041055	0.958950
C:ubiquitin ligase complex	122	4390	0.049298	0.950710
C:nucleolus	63	4449	0.052885	0.947125
P:signal transduction	994	3791	0.059572	0.940431
P:development	649	4136	0.068289	0.931715
C:endosome	33	4479	0.072924	0.927094
P:cellular morphogenesis	111	4674	0.096274	0.903738
F:DNA binding	628	4395	0.127413	0.872593
F:magnesium ion binding	135	4888	0.178351	0.821664
C:endoplasmic reticulum	268	4244	0.191479	0.808535
P:protein complex assembly	122	4663	0.220653	0.779367
F:carrier activity	149	4874	0.234886	0.765132
P:cofactor metabolism	100	4685	0.290470	0.709555
F:phosphoric ester hydrolase activity	113	4910	0.322610	0.677414
P:catabolism	260	4525	0.424096	0.575922
C:chromosome	108	4404	0.488428	0.511602
P:alcohol metabolism	133	4652	0.577437	0.422588
P:protein kinase cascade	118	4667	0.594004	0.406023
P:cell differentiation	173	4612	0.687263	0.312757
F:calcium ion binding	280	4743	0.760671	0.239342

P:amino acid metabolism	112	4673	0.782782	0.217238
P:organ development	213	4572	0.818392	0.181621
P:morphogenesis	212	4573	0.820673	0.179340
P:carbohydrate metabolism	220	4565	0.835908	0.164104
P:G-protein coupled receptor protein signaling pathway	153	4632	0.846529	0.153485
F:zinc ion binding	600	4423	0.849586	0.150421
P:negative regulation of biological process	327	4458	0.883863	0.116146
P:cell surface receptor linked signal transduction	363	4422	0.916573	0.083433
P:DNA metabolism	266	4519	0.922990	0.077016
P:cell motility	105	4680	0.951250	0.048757
F:iron ion binding	111	4912	0.978445	0.021559
F:enzyme regulator activity	238	4785	0.985550	0.014452
P:sensory perception	111	4674	0.991960	0.008042
P:cell-cell signaling	176	4609	0.993980	0.006021
P:lipid biosynthesis	101	4684	0.994426	0.005576
P:response to chemical stimulus	129	4656	0.995375	0.004626
P:positive regulation of biological process	275	4510	0.995402	0.004599
P:response to abiotic stimulus	148	4637	0.995557	0.004444
P:carboxylic acid metabolism	225	4560	0.997474	0.002527
P:apoptosis	244	4541	0.998627	0.001373
C:plasma membrane	608	3904	0.999709	0.000291
F:nuclease activity	60	4963	0.999915	8.50E-05
C:peroxisome	49	4463	0.999920	8.02E-05
C:extracellular matrix	111	4401	0.999945	5.52E-05
P:proteolysis	259	4526	0.999955	4.53E-05
C:mitochondrion	398	4114	0.999970	3.00E-05
P:cell adhesion	242	4543	0.999998	2.16E-06
P:cell proliferation	258	4527	0.999998	1.65E-06
F:peptidase activity	227	4796	0.999999	6.15E-07
C:lysosome	77	4435	1.000000	6.38E-08
P:electron transport	151	4634	1.000000	7.64E-10
P:lipid metabolism	260	4525	1.000000	5.95E-11
F:receptor binding	221	4802	1.000000	2.15E-11
F:receptor activity	391	4632	1.000000	1.11E-11
F:oxidoreductase activity	309	4714	1.000000	2.35E-12
P:response to stress	446	4339	1.000000	5.05E-26
C:extracellular space	179	4333	1.000000	3.49E-37
P:immune response	270	4515	1.000000	1.16E-44
P:response to biotic stimulus	318	4467	1.000000	4.08E-49
