# Supplementary material for: Weak correlation between sequence conservation in promoter regions and in protein-coding regions of human-mouse orthologous gene pairs
Source: BMC Genomics. 2008 Apr 2;9:152. doi: 10.1186/1471-2164-9-152 (PMC2335122; doi:10.1186/1471-2164-9-152)
Supplement: Additional file 6 — Distribution of percentage identities of human and mouse protein sequences for GO categories with significant tendencies. A. GO categories with high protein conservation, B. GO categories with low protein conservation. [file 1471-2164-9-152-S6.pdf]

Additional file 6

A

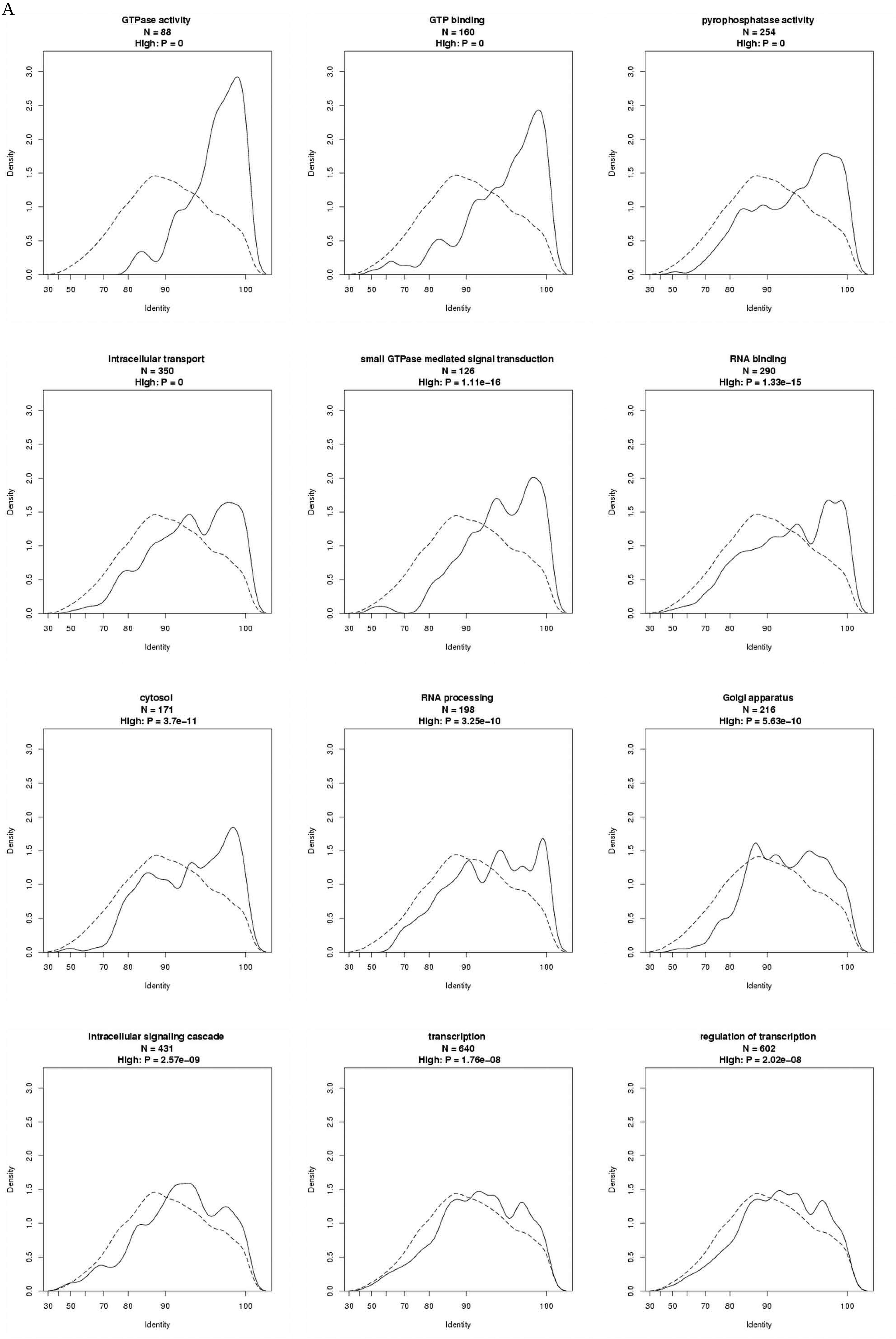

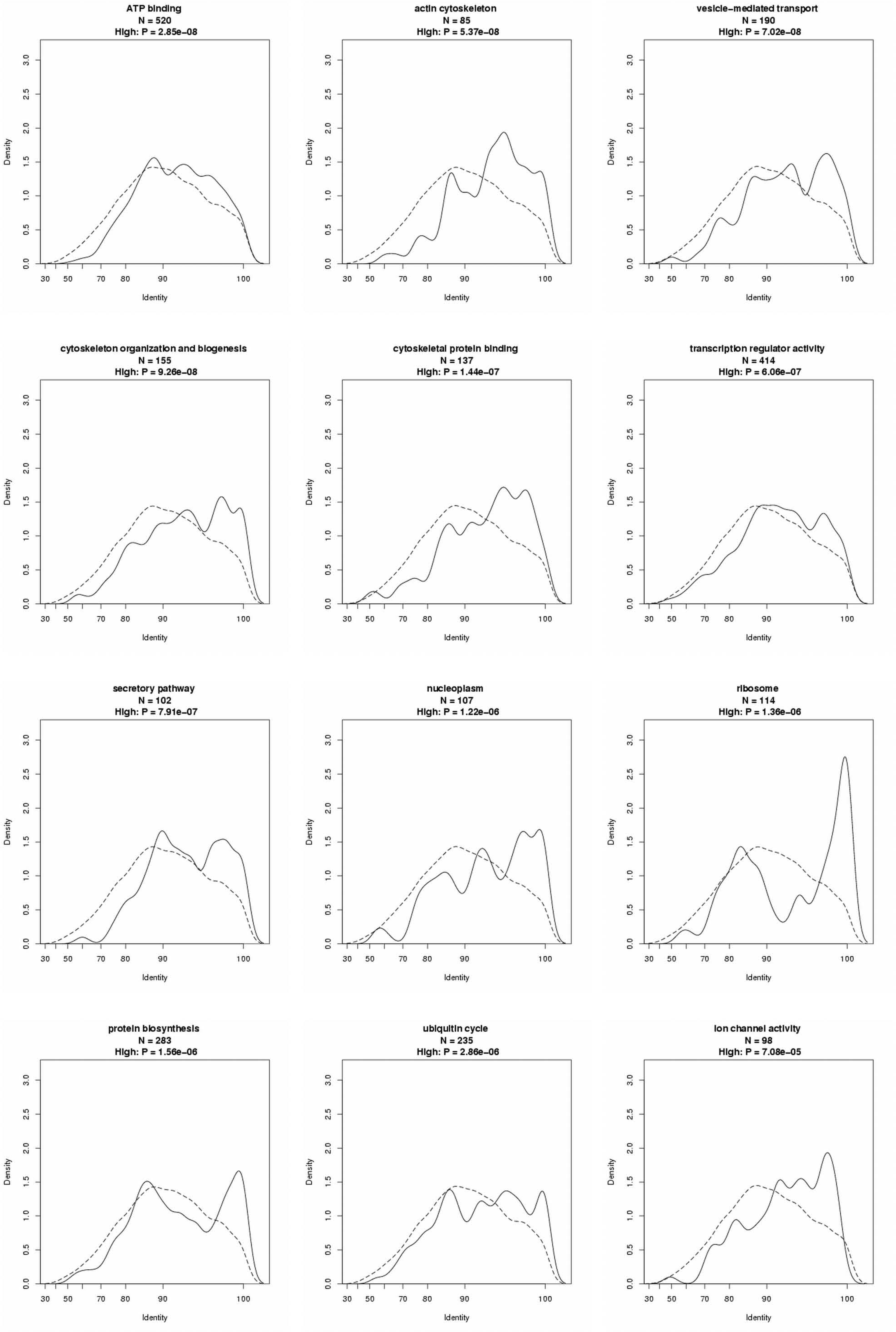

ATP binding
N = 520
High: P = 2.85e−08
actin cytoskeleton
N = 85
High: P = 5.37e−08
vesicle−mediated transport
N = 190
High: P = 7.02e−08
cytoskeleton organization and biogenesis
N = 155
High: P = 9.26e−08
cytoskeletal protein binding
N = 137
High: P = 1.44e−07
transcription regulator activity
N = 414
High: P = 6.06e−07
secretory pathway
N = 102
High: P = 7.91e−07
nucleoplasm
N = 107
High: P = 1.22e−06
ribosome
N = 114
High: P = 1.36e−06
protein biosynthesis
N = 283
High: P = 1.56e−06
ubiquitin cycle
N = 235
High: P = 2.86e−06
ion channel activity
N = 98
High: P = 7.08e−05
Density
Identity
0.0
0.5
1.0
1.5
2.0
2.5
3.0
30
50
70
80
90
100

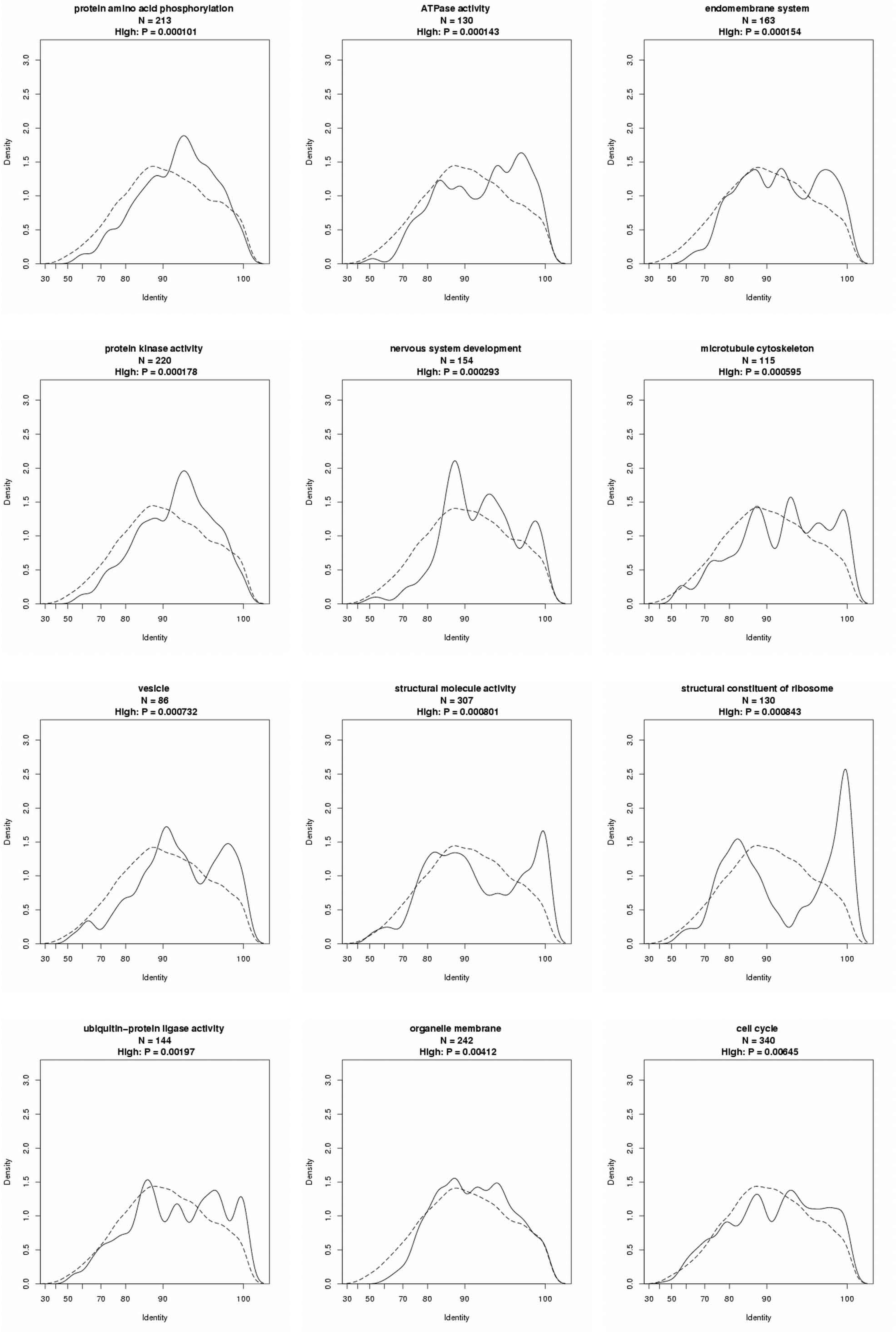
protein amino acid phosphorylation
N = 213
High: P = 0.000101
ATPase activity
N = 130
High: P = 0.000143
endomembrane system
N = 163
High: P = 0.000154
protein kinase activity
N = 220
High: P = 0.000178
nervous system development
N = 154
High: P = 0.000293
microtubule cytoskeleton
N = 115
High: P = 0.000595
vesicle
N = 86
High: P = 0.000732
structural molecule activity
N = 307
High: P = 0.000801
structural constituent of ribosome
N = 130
High: P = 0.000843
ubiquitin-protein ligase activity
N = 144
High: P = 0.00197
organelle membrane
N = 242
High: P = 0.00412
cell cycle
N = 340
High: P = 0.00645
Density
Identity
0.0 0.5 1.0 1.5 2.0 2.5 3.0
30 50 70 80 90 100

B

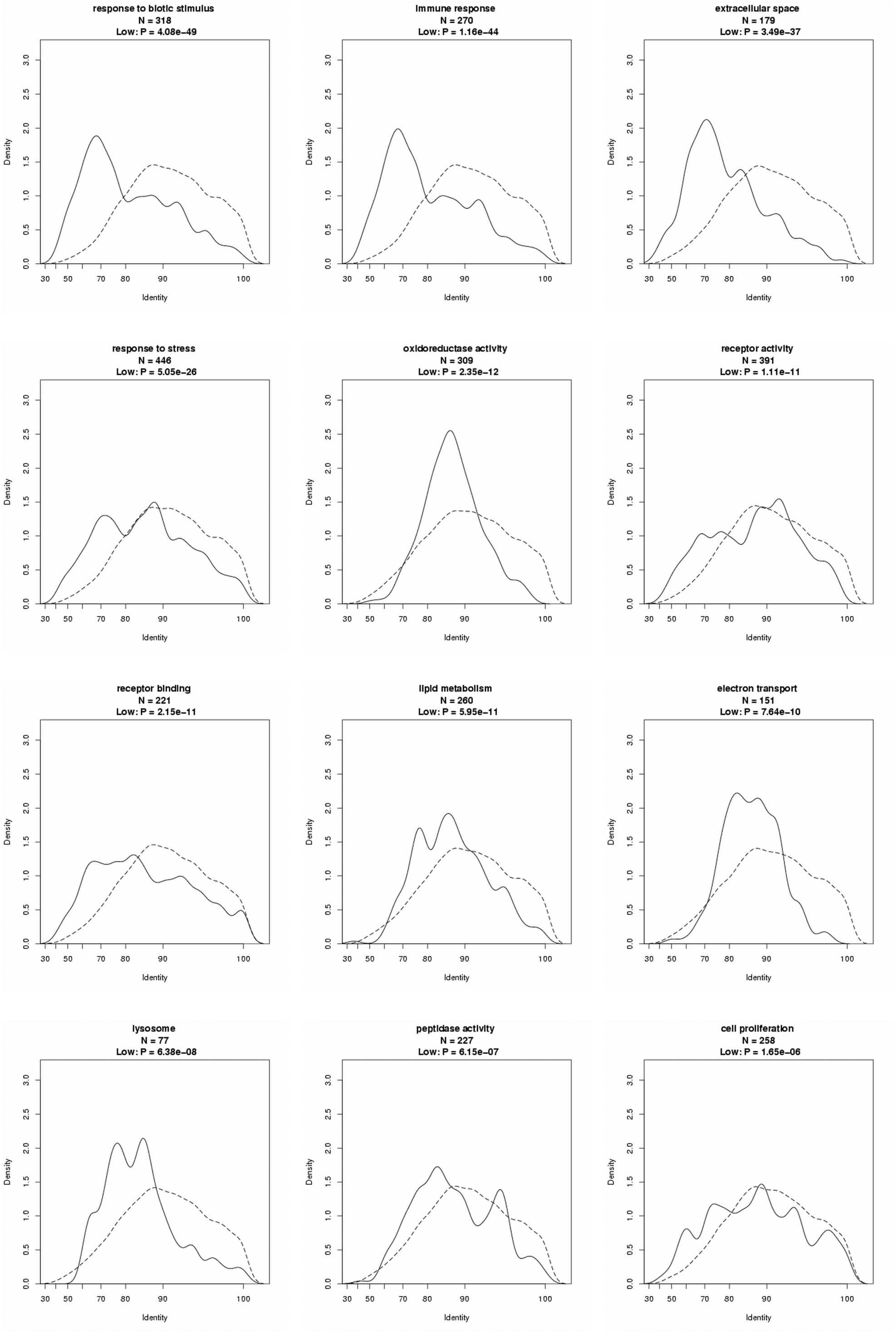
response to biotic stimulus
N = 318
Low: P = 4.08e−49
immune response
N = 270
Low: P = 1.16e−44
extracellular space
N = 179
Low: P = 3.49e−37
response to stress
N = 446
Low: P = 5.05e−26
oxidoreductase activity
N = 309
Low: P = 2.35e−12
receptor activity
N = 391
Low: P = 1.11e−11
receptor binding
N = 221
Low: P = 2.15e−11
lipid metabolism
N = 260
Low: P = 5.95e−11
electron transport
N = 151
Low: P = 7.64e−10
lysosome
N = 77
Low: P = 6.38e−08
peptidase activity
N = 227
Low: P = 6.15e−07
cell proliferation
N = 258
Low: P = 1.65e−06
Density
Identity
0.0
0.5
1.0
1.5
2.0
2.5
3.0
30
50
70
80
90
100

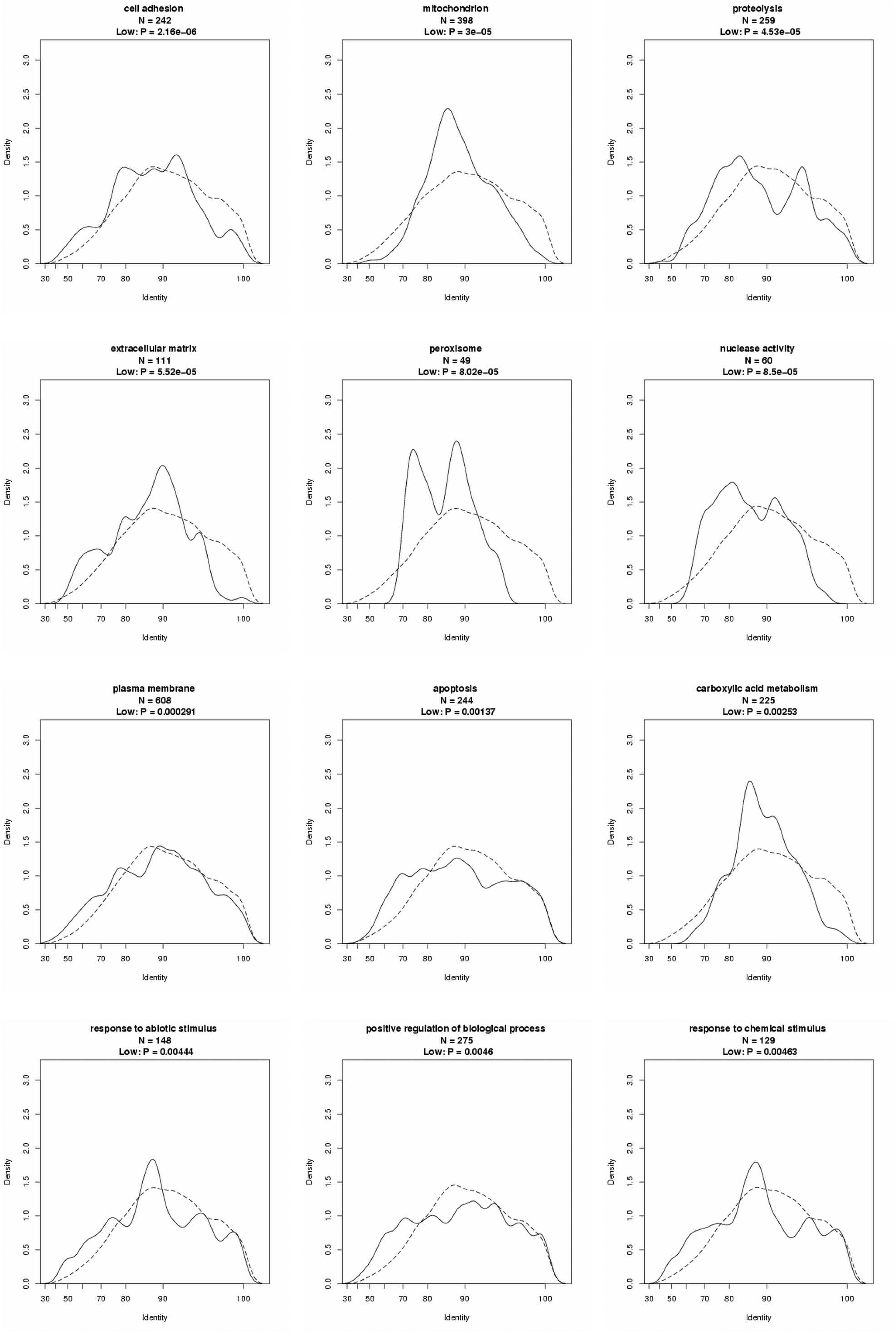
cell adhesion
N = 242
Low: P = 2.16e−06
mitochondrion
N = 398
Low: P = 3e−05
proteolysis
N = 259
Low: P = 4.53e−05
extracellular matrix
N = 111
Low: P = 5.52e−05
peroxisome
N = 49
Low: P = 8.02e−05
nuclease activity
N = 60
Low: P = 8.5e−05
plasma membrane
N = 608
Low: P = 0.000291
apoptosis
N = 244
Low: P = 0.00137
carboxylic acid metabolism
N = 225
Low: P = 0.00253
response to abiotic stimulus
N = 148
Low: P = 0.00444
positive regulation of biological process
N = 275
Low: P = 0.0046
response to chemical stimulus
N = 129
Low: P = 0.00463
Density
0.0 0.5 1.0 1.5 2.0 2.5 3.0
Identity
30 50 70 80 90 100

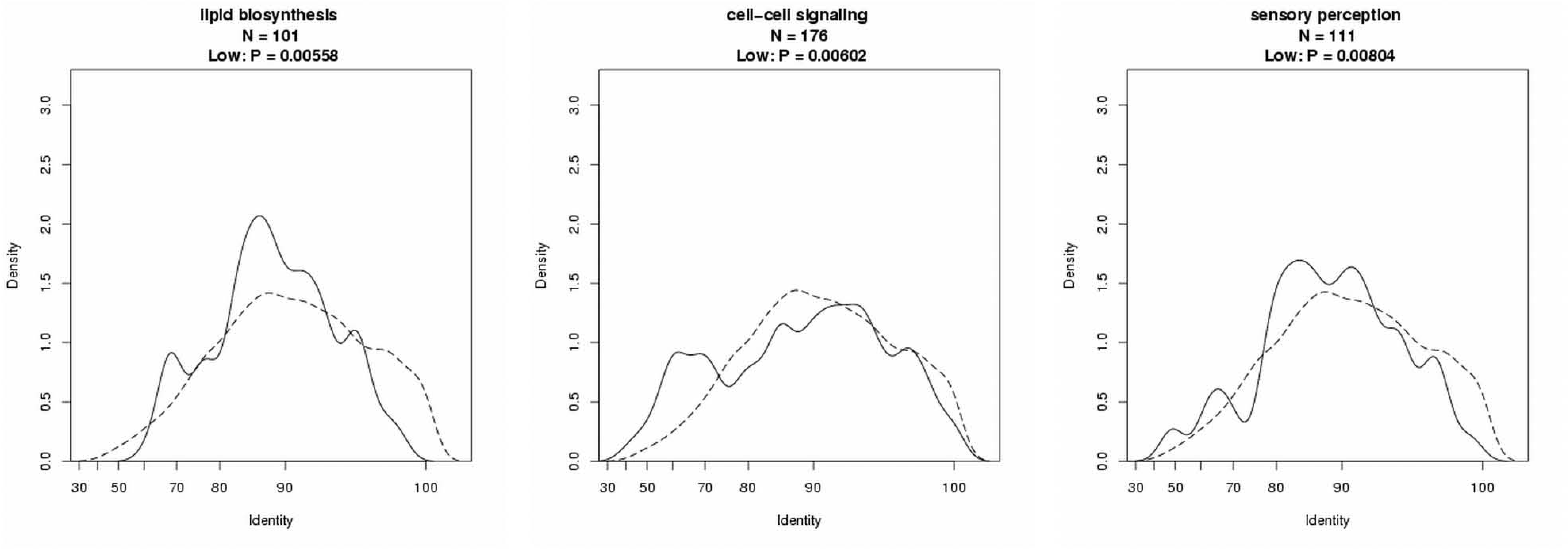

lipid biosynthesis
N = 101
Low: P = 0.00558
cell-cell signaling
N = 176
Low: P = 0.00602
sensory perception
N = 111
Low: P = 0.00804
Density
Identity
0.0
0.5
1.0
1.5
2.0
2.5
3.0
30
50
70
80
90
100
