# Supplementary figures and images for: Weak correlation between sequence conservation in promoter regions and in protein-coding regions of human-mouse orthologous gene pairs
Source: BMC Genomics. 2008 Apr 2;9:152. doi: 10.1186/1471-2164-9-152 (PMC2335122; doi:10.1186/1471-2164-9-152)

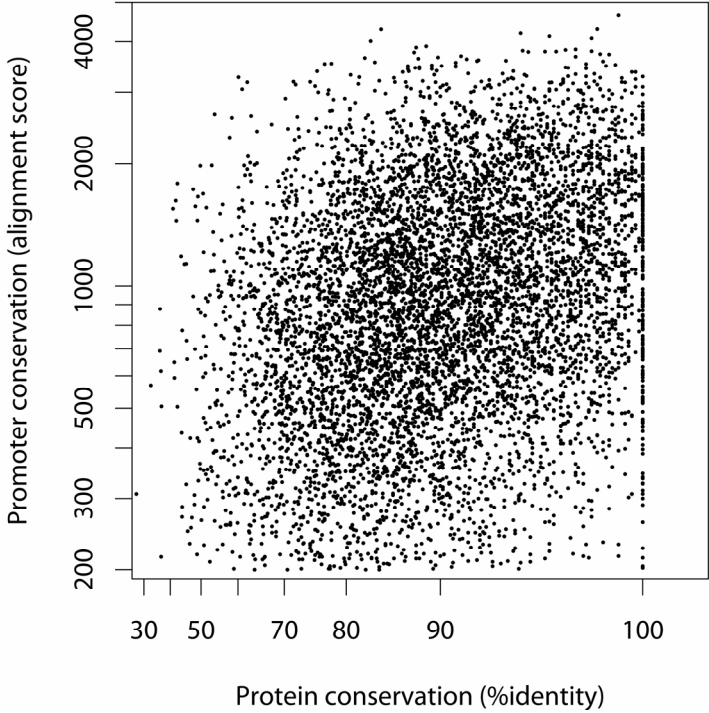

Supplement: Additional file 7 — Scatter plot of protein conservation and promoter conservation for human and mouse orthologous genes. [file 1471-2164-9-152-S7.pdf]

Additional file 8

A. human

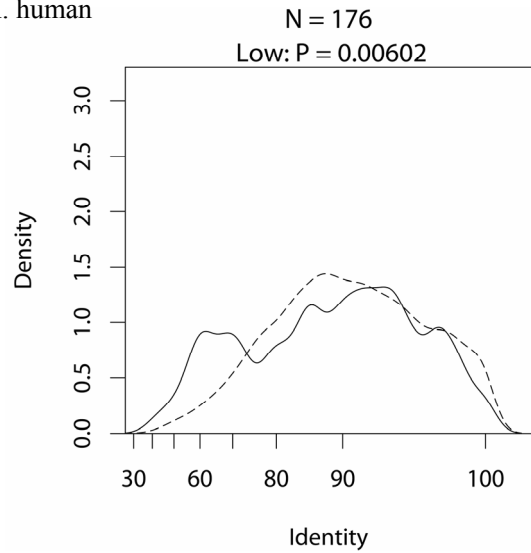

B. mouse

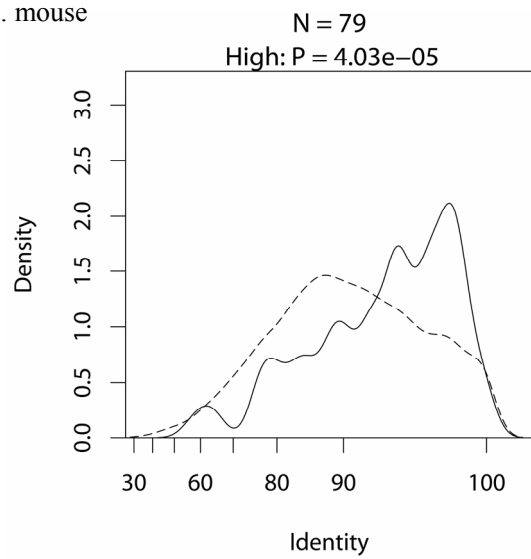

C.

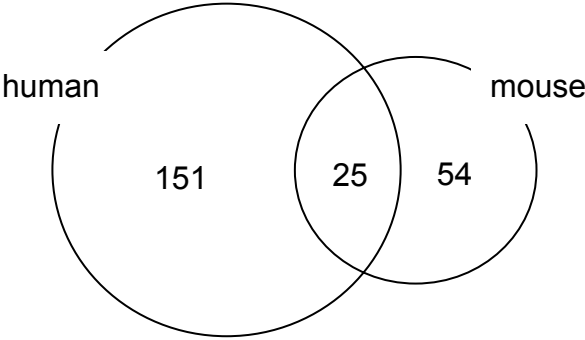

Supplement: Additional file 8 — Protein conservation of human and mouse 'cell-cell signaling' genes. [file 1471-2164-9-152-S8.pdf]
