# Supplementary material for: Weak correlation between sequence conservation in promoter regions and in protein-coding regions of human-mouse orthologous gene pairs
Source: BMC Genomics. 2008 Apr 2;9:152. doi: 10.1186/1471-2164-9-152 (PMC2335122; doi:10.1186/1471-2164-9-152)
Supplement: Additional file 9 — Protein conservations and RefSeq annotations for 'ribosome' category. Genes are sorted by the percentage identity. [file 1471-2164-9-152-S9.pdf]

Additional file 9

%identity	RefSeq annotation
100.0	ribosomal protein L11
100.0	ribosomal protein L15
100.0	ribosomal protein L23
100.0	ribosomal protein L23a
100.0	ribosomal protein L24
100.0	ribosomal protein L26
100.0	ribosomal protein L27
100.0	ribosomal protein L30
100.0	ribosomal protein L31
100.0	ribosomal protein L36a
100.0	ribosomal protein L37
100.0	ribosomal protein L39
100.0	ribosomal protein L8
100.0	ribosomal protein S11
100.0	ribosomal protein S13
100.0	ribosomal protein S14
100.0	ribosomal protein S15
100.0	ribosomal protein S15a
100.0	ribosomal protein S18
100.0	ribosomal protein S20
100.0	ribosomal protein S23
100.0	ribosomal protein S27 (metallopanstimulin 1)
100.0	ribosomal protein S29
100.0	ribosomal protein S4, X-linked
100.0	ribosomal protein S7
99.6	eukaryotic translation initiation factor 2, subunit 3 gamma, 52kDa
99.6	ribosomal protein S3
99.5	ribosomal protein L10
99.5	ribosomal protein L10a
99.5	ribosomal protein L19
99.4	ribosomal protein L21
99.4	ribosomal protein S27a
99.3	ribosomal protein S19
99.2	ribosomal protein L22
99.1	ribosomal protein L35a
99.0	ribosomal protein L9
99.0	ribosomal protein SA
98.8	chromosome 15 open reading frame 15
98.8	ribosomal protein S10
98.6	ribosomal protein L38
98.6	ribosomal protein S2
98.5	ribosomal protein L28
98.5	ribosomal protein S5
98.4	ribosomal protein L35
98.3	ribosomal protein L3
98.3	ribosomal protein L5
97.9	ribosomal protein S16
97.7	Finkel-Biskis-Reilly murine sarcoma virus (FBR-MuSV) ubiquitously expressed (fox derived); ribosomal protein S30
97.5	dynamin 3
97.5	ribosomal protein, large, P0
97.4	ribosomal protein, large, P1
97.3	ribosomal protein L27a
96.6	ADP-ribosylation factor-like 6 interacting protein 1
95.9	ribosomal protein L7
95.6	ribosomal protein L13a
95.5	eukaryotic translation initiation factor 2, subunit 2 beta, 38kDa
95.5	signal recognition particle 68kDa
95.4	splicing factor 1
95.2	ribosomal protein L36
95.2	ribosomal protein S21
94.3	APEX nuclease (multifunctional DNA repair enzyme) 1
94.1	fragile X mental retardation 1
92.8	formin binding protein 1

91.9	ribosomal protein L4
91.1	mitochondrial ribosomal protein S16
90.8	mitochondrial ribosomal protein S21
90.5	mitochondrial ribosomal protein S25
89.3	mitochondrial ribosomal protein L13
89.2	intraflagellar transport 88 homolog (Chlamydomonas)
88.9	mitochondrial ribosomal protein L39
88.8	mitochondrial ribosomal protein L22
88.6	ribosomal protein L6
88.4	phytoceramidase, alkaline
88.3	queuine tRNA-ribosyltransferase 1 (tRNA-guanine transglycosylase)
87.7	megalencephalic leukoencephalopathy with subcortical cysts 1
87.5	mitochondrial ribosomal protein L11
87.0	mitochondrial ribosomal protein L24
86.9	mitochondrial ribosomal protein S10
86.7	mitochondrial ribosomal protein S14
86.3	mitochondrial ribosomal protein S17
85.6	mitochondrial ribosomal protein S6
85.5	mitochondrial ribosomal protein S12
85.1	mitochondrial ribosomal protein L17
84.6	mitochondrial ribosomal protein L12
84.3	mitochondrial ribosomal protein S36
84.3	mitochondrial ribosomal protein S7
83.8	mitochondrial ribosomal protein S24
83.6	ribosomal protein L7-like 1
83.5	mitochondrial ribosomal protein L30
83.1	mitochondrial ribosomal protein L27
83.1	mitochondrial ribosomal protein L49
83.0	mitochondrial ribosomal protein L23
82.7	mitochondrial ribosomal protein L9
82.2	mitochondrial ribosomal protein L3
82.1	mitochondrial ribosomal protein L21
82.1	mitochondrial ribosomal protein S33
81.1	mitochondrial ribosomal protein S18A
80.1	ribosomal protein L29
79.9	acyl-Coenzyme A binding domain containing 5
79.9	mitochondrial ribosomal protein S5
78.9	mitochondrial ribosomal protein S22
78.2	mitochondrial ribosomal protein L1
77.8	mitochondrial ribosomal protein S18B
77.0	mitochondrial ribosomal protein L47
77.0	mitochondrial ribosomal protein S9
76.4	mitochondrial ribosomal protein L42
75.0	mitochondrial ribosomal protein L34
74.8	mitochondrial ribosomal protein L40
73.9	mitochondrial ribosomal protein S30
72.7	mitochondrial ribosomal protein S28
72.2	mitochondrial ribosomal protein S11
63.6	mitochondrial ribosomal protein S15
59.2	mitochondrial ribosomal protein L36
56.2	ribosomal L1 domain containing 1
