# Supplementary material for: Weak correlation between sequence conservation in promoter regions and in protein-coding regions of human-mouse orthologous gene pairs
Source: BMC Genomics. 2008 Apr 2;9:152. doi: 10.1186/1471-2164-9-152 (PMC2335122; doi:10.1186/1471-2164-9-152)
Supplement: Additional file 10 — Promoter conservations and RefSeq annotations for 'ribosome' category. Genes are sorted by the alignment score. [file 1471-2164-9-152-S10.pdf]

Additional file 10

Alignment score	Refseq annotation
2455	mitochondrial ribosomal protein S18B
2419	ribosomal protein S29
2242	mitochondrial ribosomal protein S12
2212	ribosomal protein L7
1955	mitochondrial ribosomal protein L49
1954	ribosomal protein S18
1841	ribosomal protein S19
1835	splicing factor 1
1615	mitochondrial ribosomal protein L40
1551	mitochondrial ribosomal protein L13
1468	mitochondrial ribosomal protein S11
1459	ribosomal protein L36a
1403	eukaryotic translation initiation factor 2, subunit 2 beta, 38kDa
1268	ribosomal protein L4
1242	megalencephalic leukoencephalopathy with subcortical cysts 1
1202	ribosomal protein S27a
1173	mitochondrial ribosomal protein L17
1142	mitochondrial ribosomal protein L11
1141	ribosomal protein L22
1141	ribosomal protein S27 (metallopanstimulin 1)
1123	ribosomal protein L10
1121	fragile X mental retardation 1
1113	mitochondrial ribosomal protein S7
1074	Finkel-Biskis-Reilly murine sarcoma virus (FBR-MuSV) ubiquitously expressed (fox derived); ribosomal protein S30
1031	ribosomal protein S16
1028	dynamin 3
1017	signal recognition particle 68kDa
993	mitochondrial ribosomal protein S6
970	ribosomal protein L19
945	ribosomal protein L13a
927	mitochondrial ribosomal protein L27
902	ribosomal protein L27
900	ribosomal protein, large, P1
896	acyl-Coenzyme A binding domain containing 5
889	eukaryotic translation initiation factor 2, subunit 3 gamma, 52kDa
886	phytoceramidase, alkaline
877	ribosomal protein L35a
848	ribosomal protein S14
846	ribosomal protein L30
834	mitochondrial ribosomal protein S21
824	mitochondrial ribosomal protein L1
823	mitochondrial ribosomal protein L3
820	ribosomal protein S4, X-linked
813	formin binding protein 1
809	mitochondrial ribosomal protein L30
791	ADP-ribosylation factor-like 6 interacting protein 1
787	ribosomal protein L15
782	ribosomal protein L9
774	queuine tRNA-ribosyltransferase 1 (tRNA-guanine transglycosylase)
742	ribosomal protein S23
738	mitochondrial ribosomal protein L47
731	ribosomal protein S3
726	ribosomal protein S2
717	ribosomal protein L23a
703	ribosomal protein L39
702	ribosomal protein L5
698	ribosomal protein L26
694	mitochondrial ribosomal protein S36
686	ribosomal protein L11
673	ribosomal protein L21
672	mitochondrial ribosomal protein S18A
671	ribosomal protein S13

669	ribosomal protein L29
666	ribosomal protein S5
657	ribosomal protein S7
646	mitochondrial ribosomal protein L24
643	mitochondrial ribosomal protein S30
640	ribosomal protein L7-like 1
631	ribosomal protein S20
628	ribosomal protein L38
626	ribosomal protein S10
623	ribosomal protein L6
618	ribosomal protein L37
607	ribosomal protein L23
593	mitochondrial ribosomal protein L42
580	mitochondrial ribosomal protein S28
573	mitochondrial ribosomal protein S15
571	mitochondrial ribosomal protein L34
550	mitochondrial ribosomal protein S33
548	mitochondrial ribosomal protein S22
545	ribosomal protein L24
536	chromosome 15 open reading frame 15
528	ribosomal protein L3
524	ribosomal protein L31
522	mitochondrial ribosomal protein S24
516	ribosomal protein L28
516	ribosomal protein L35
514	mitochondrial ribosomal protein S10
510	intraflagellar transport 88 homolog (Chlamydomonas)
504	ribosomal protein, large, P0
498	mitochondrial ribosomal protein S14
491	mitochondrial ribosomal protein L23
486	APEX nuclease (multifunctional DNA repair enzyme) 1
481	ribosomal protein L27a
475	mitochondrial ribosomal protein L9
472	mitochondrial ribosomal protein L22
465	ribosomal protein S15a
448	mitochondrial ribosomal protein S9
439	mitochondrial ribosomal protein L21
421	ribosomal protein S15
405	ribosomal protein SA
398	ribosomal protein L10a
356	mitochondrial ribosomal protein L12
350	mitochondrial ribosomal protein S5
314	ribosomal protein S11
306	mitochondrial ribosomal protein S25
302	ribosomal L1 domain containing 1
300	ribosomal protein L36
297	ribosomal protein S21
289	ribosomal protein L8
283	mitochondrial ribosomal protein L39
247	mitochondrial ribosomal protein S17
245	mitochondrial ribosomal protein L36
223	mitochondrial ribosomal protein S16
