# Supplementary material for: Weak correlation between sequence conservation in promoter regions and in protein-coding regions of human-mouse orthologous gene pairs
Source: BMC Genomics. 2008 Apr 2;9:152. doi: 10.1186/1471-2164-9-152 (PMC2335122; doi:10.1186/1471-2164-9-152)
Supplement: Additional file 11 — Promoter conservation tendency for each GO category based on alignment length and percentage identity. [file 1471-2164-9-152-S11.pdf]

Additional file 11

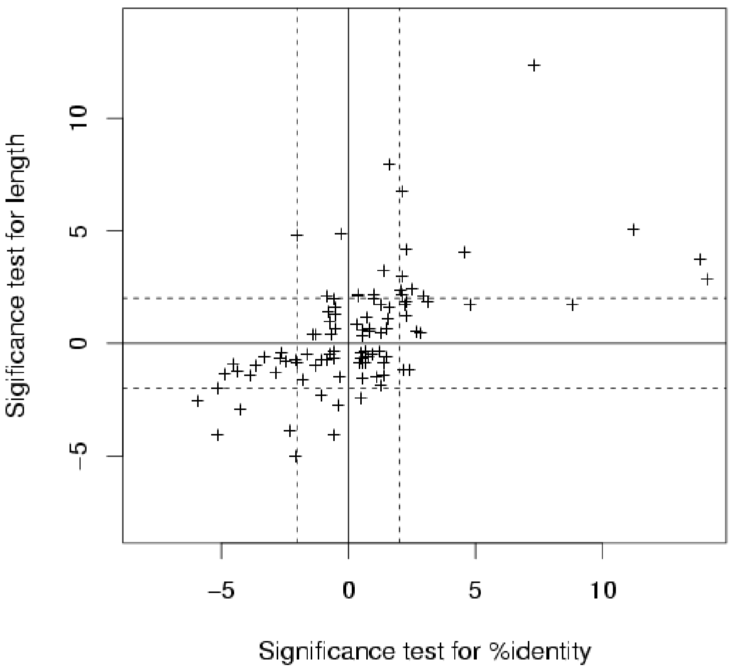


Each axis is based on the common logarithm of P-values of significance tests for each GO. Plus means high identity and minus means low identity. Dashed lines correspond to a P-value of 0.01.
